# Accounting for variability when resurrecting dormant propagules substantiates their use in eco‐evolutionary studies

**DOI:** 10.1111/eva.13316

**Published:** 2021-11-27

**Authors:** Megan L. Vahsen, Rachel M. Gentile, Jennifer L. Summers, Helena S. Kleiner, Benjamin Foster, Regina M. McCormack, Evan W. James, Rachel A. Koch, Dailee L. Metts, Colin Saunders, James Patrick Megonigal, Michael J. Blum, Jason S. McLachlan

**Affiliations:** ^1^ Department of Biological Sciences University of Notre Dame Notre Dame Indiana USA; ^2^ Department of Ecology & Evolutionary Biology University of Tennessee Knoxville Tennessee USA; ^3^ Smithsonian Environmental Research Center Edgewater Maryland USA; ^4^ Southeast Environmental Research Center Florida International University Miami Florida USA

**Keywords:** Bayesian hierarchical modeling, germination, resurrection ecology, *Schoenoplectus americanus*, seed viability

## Abstract

There has been a steady rise in the use of dormant propagules to study biotic responses to environmental change over time. This is particularly important for organisms that strongly mediate ecosystem processes, as changes in their traits over time can provide a unique snapshot into the structure and function of ecosystems from decades to millennia in the past. Understanding sources of bias and variation is a challenge in the field of resurrection ecology, including those that arise because often‐used measurements like seed germination success are imperfect indicators of propagule viability. Using a Bayesian statistical framework, we evaluated sources of variability and tested for zero‐inflation and overdispersion in data from 13 germination trials of soil‐stored seeds of *Schoenoplectus americanus*, an ecosystem engineer in coastal salt marshes in the Chesapeake Bay. We hypothesized that these two model structures align with an ecological understanding of dormancy and revival: zero‐inflation could arise due to failed germinations resulting from inviability or failed attempts to break dormancy, and overdispersion could arise by failing to measure important seed traits. A model that accounted for overdispersion, but not zero‐inflation, was the best fit to our data. Tetrazolium viability tests corroborated this result: most seeds that failed to germinate did so because they were inviable, not because experimental methods failed to break their dormancy. Seed viability declined exponentially with seed age and was mediated by seed provenance and experimental conditions. Our results provide a framework for accounting for and explaining variability when estimating propagule viability from soil‐stored natural archives which is a key aspect of using dormant propagules in eco‐evolutionary studies.

## 1 INTRODUCTION

Reviving dormant propagules can be a powerful approach for studying biotic responses to global change (Weider et al., 2018). This approach is often referred to as ‘resurrection ecology,’ and it serves as a lens for examining genetic and phenotypic change over time, for inferring processes underlying responses to stressor exposure (Brendonck & De Meester, 2003; Burge et al., 2018; Weider et al., 2018), and for lending insight into climatic and ecosystem processes across historical time (Burge et al., 2018). Foundational resurrection ecology research provided empirical evidence of rapid evolution in short‐lived zooplankton (De Meester et al., 2011; Frisch et al., 2014; Geerts et al., 2015; Hairston et al., 1999; Kerfoot et al., 1999). More recently, persistent soil‐stored seed banks have become recognized as a promising resource for reconstructing records of plant responses to environmental change (Blum et al., 2021; Fennell et al., 2014; Summers et al., 2018). Seeds recovered from time‐stratified soils and sediments have long served as proxy records of past geological‐ and climate‐related conditions such as relative sea‐level rise (e.g., Jarrell et al., 2016; Saunders, 2003; Saunders et al., 2006; Törnqvist et al., 2004). Soil‐stored seeds, especially from sedges and rushes, are now increasingly being revived to gain insight into demographic and genetic variation over time (Bennington et al., 1991; Fennell et al., 2014; Gugerli et al., 2005; McGraw, 1993; Summers et al., 2018; Vavrek et al., 1991). Importantly, traits of sedges and rushes are tightly linked to biogeochemical processes such as carbon sequestration (Kirwan & Megonigal, 2013; Langley & Megonigal, 2010), so understanding their trait variation across time also lends essential insight into broader ecosystem processes from earlier decades to centuries. Despite these advances and the potential implications of this work, the use of soil‐stored seed banks for eco‐evolutionary studies is still limited (Blum et al., 2021; Etterson et al., 2016; Franks et al., 2008), partly due to concerns of biased representation that are common within the field of resurrection ecology (Brendonck & De Meester, 2003; Hairston & Kearns, 2002; Weis, 2018). Therefore, understanding and constraining uncertainty around propagule viability and revival could help allay concerns about biased representation (Summers et al., 2018).

Seminal agricultural and ecological studies of seed germination and dormancy (Biere, 1991; Chouard, 1960; Heydeker, 1977; Kalisz, 1989; Mayer & Poljakoff‐Mayber, 1982; Srivastava, 2002) have demonstrated that several factors can result in biased representation. For example, a nonrandom subset of seeds that fall to the ground may enter the seed bank (Franks et al., 2018; Templeton & Levin, 1979; Weis, 1982). Bias might also arise from progressive attrition (i.e., mortality), where viability (and thus revival) declines with time since burial (Hairston et al., 1996; Summers et al., 2018; Weis, 2018). Nonrandom attrition can occur if, for example, seeds exhibit differences in traits like size or coat thickness that influence the likelihood of persistence (Bakker et al., 1996; Mohamed‐Yasseen et al., 1994; Schwienbacher et al., 2010), or if there is pre‐emergence selection acting on traits that covary with germination or seed viability (Weis, 2018). Similarly, biased representation in ‘resurrected’ populations might arise if experimental approaches result in nonrandom germination (i.e., apparent bias due to artificial selection). Random variation that is unrelated to seed traits or experimental design may also affect seed revival. Finally, as germinated seeds usually represent only a small subset of the corresponding historical population, accounting for sampling error in subsequent experiments could be important in estimating uncertainty in evolutionary change over time. Disentangling potential sources of bias and variation could substantially advance understanding of how seed germination data can serve as a proxy estimate of viability and thus improve our understanding of how dormant propagules can be used for eco‐evolutionary studies.

Bias can overall be minimized by using study species with large propagule population sizes, reducing the likelihood of uneven sampling and false signatures of selection (Brendonck & De Meester, 2003; Weider et al., 1997). Using study species that produce highly resilient propagules can also reduce bias (e.g., Blum et al., 2021; Summers et al., 2018). This is well‐reflected in paleoecological studies of coastal marshes, which have frequently focused on sedges that produce large crops of seeds with durable coats capable of persisting in marsh soils for up to millennia (Brush, 2001; Jarrell et al., 2016; Miller et al., 1997; Saunders, 2003; Sherfy & Kirkpatrick, 1999; Törnqvist et al., 2004). Notably, prior work with a century‐long *Schoenoplectus americanu*s seed bank demonstrated that some potential drawbacks could be overcome (Summers et al., 2018). In that study, seeds of *S*. *americanus* from five stratified soil layers spanning approximately 100 years demonstrated genotypic differences among different age cohorts and between age cohorts and extant plants. Genetic diversity (i.e., allelic richness, heterozygosity) based on microsatellite genotyping did not decline with depth, suggesting that the observed pattern of differentiation is likely not due to attrition. Despite these findings, there remain outstanding questions about the fraction of seeds recovered from the *S*. *americanus* seed bank that failed to germinate. For example, it is unknown if the seeds that fail to germinate are inviable, if methods to break dormancy fail, and how seed age mediates these processes. Further, the extent to which traits related to seed dormancy are correlated with those of adult plants is unknown, and this is critical to the understanding of whether cohorts of resurrected propagules are representative of historical populations (Bennington & McGraw, 1995).

Even when a large number of durable propagules are available for study as in Summers et al. (2018), estimating seed viability can be difficult. Destructive assays like tetrazolium tests that register cellular respiration (Santos et al., 2007) can deliver valuable perspectives on seed viability but prevent subsequent use (e.g., for constructing experimental populations). Estimates of the probability of germination success from germination trials used to “resurrect” dormant propagules thus serve as imperfect proxy measurements of seed viability and allow for germinated propagules to be used in further study. We suggest that post hoc hierarchical statistical modeling (e.g., Hobbs & Hooten, 2015) can help identify and differentiate some of the sources of variability and bias from experimental data on proxy measures of viability like germination success. For example, we can use statistical models to better understand how much our estimates of germination rate vary due to experimental methods indicating how generalizable viability estimates are for a species or population.

Thus far, the process of viability decay in persistent soil‐stored seedbanks has largely been characterized under artificial or seminatural conditions (Burnside et al., 1986; Kilivaan & Bandurski, 1981) like short‐term burial experiments (e.g., Schütz, 2000). Some mathematical modeling (Cohen, 1966) has also provided insight into tradeoffs (e.g., germination vs. storage) that can influence seed burial, persistence, and viability. Empirical estimation of *in situ* viability decay can thus offer valuable, and arguably more realistic, perspectives on how seeds persist under natural conditions (Fennell et al., 2014; Summers et al., 2018). Moreover, because it can yield insight into the merits of different methods taken to elicit germination of dormant seeds, empirical estimation of *in situ* viability using statistical models can be useful for testing hypotheses about the likelihood, magnitude and source(s) of variability, and bias due to experimental conditions. Linking understanding of seed banking ecology with the design of statistical models can provide even greater insight. For example, bias in seed germination data can result because seeds can fail to germinate (i.e., zeros in the data) as a result of inviability or because attempts at revival failed to break dormancy. Zero‐inflated statistical models, which are often used to account for zeros that arise from separate processes (Hooten & Hefley, 2019) might therefore be a reasonable choice of statistical likelihood for fitting germination trial data to improve understanding of the process(es) underlying germination failures.

In this study, we evaluated the *in situ* viability of soil‐stored seeds using germination trial data from *S*. *americanus*, a dominant sedge that serves as a model species for studying plant and coastal marsh ecosystem responses to global change (Drake, 2014). Previous research from Summers et al. (2018) suggests that germination success of soil‐stored *S*. *americanus* seeds declines with seed depth. However, it is still unclear whether this is because seeds become increasingly inviable with depth, if experimental methods to break dormancy fail at increasing rates with depth, or both.

Here, we use seed germination data from more than a dozen experimental assays to characterize seed viability and develop hierarchical Bayesian models to account for and explain variation in germination success related to seed age, seed provenance, and experimental conditions. We used a model selection approach to assess the merits of four possible statistical models factorially, for which we either included or did not include model components that account for zero‐inflation and overdispersion in our data (Table 1). This design allowed us to test hypotheses motivated by ecological understanding of how biases can influence germination success. First, we hypothesized that a zero‐inflated model might best fit our data because both seed mortality and an inability to break dormancy of viable seeds can lead to germination failure and give rise to an over‐representation of zeros in experimental data (Table 1; Figure S3). We evaluated the alternative hypothesis that a model accounting for overdispersion might best fit our data because key covariates, such as those related to seed quality (e.g., seed size, seed coat thickness) or the environmental conditions in which the seeds were buried, were not accounted for in our analysis (Table 1). We used the best fit model to test the coupled hypothesis that viability and germination were nonrandom due to differences in age, seed provenance, and experimental methods taken to break dormancy. Finally, we confronted our model selection results with data from tetrazolium tests of seed viability as a means to support whether or not seed germination success serves as an adequate proxy for seed viability, or similarly, if our success breaking dormancy changed as a function of seed age.

**TABLE 1:**
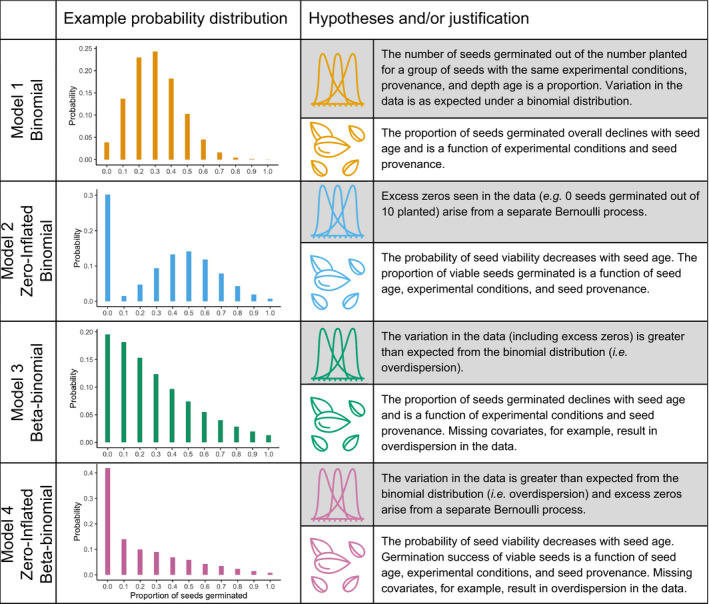
Description of statistical models including underlying hypotheses and reasoning. Example probability distributions are shown for each of the four models on the left with the number of seeds planted equal to 10. Parameter values for the distributions were chosen to accentuate differences in the shapes of the probability distributions. For each model, the hypotheses/justification for the statistical model specification are written (gray background) above the biological hypotheses/justification (white background)

Through this approach, we can assess bias and variability that may arise from experimental conditions, those that may arise due to pools of viable and inviable seeds of varying ages failing to germinate, and importantly, from using germination trial data as a proxy for seed viability. Our results provide practical guidance for reviving soil‐stored seeds for reconstructing decadal to century‐long records of plant responses to environmental change by illustrating the magnitude and direction of treatment effects on germination success (Dagne, 2004; Lambert, 1992) and demonstrating the ability for germination success to serve as a proxy for seed viability. Our analysis does not account for important sources of bias and variation that dictate how representative “resurrected” plants are of their respective historical cohorts, which would require information on how correlated traits related to seed dormancy are to traits of adult plants. Instead, we provide a framework for assessing some biases for which we have germination trial and tetrazolium viability test data to inform.

## 2 METHODS

### 2.1 Study species

The C_3_ sedge *S*. *americanus* (previously known as *Scirpus olneyi*) has been the focus of foundational research on coastal marsh responses to global environmental change including elevated atmospheric CO_2_, nutrient loading, warming, and biological invasions (e.g., Drake, 2014; Langley & Megonigal, 2010; Langley et al., 2013; Lu et al., 2019; Noyce et al., 2019). Along the Atlantic and Gulf coasts of North America, *S*. *americanus* is often the dominant plant in brackish marshes where salinity varies between 3.5 and 10 ppt (Smith, 1995). Reproduction in *S*. *americanus* involves both asexual (i.e., vegetative tillering) and sexual reproduction. *S*. *americanus* produces semispherical seeds with durable coats (Miller et al., 1997; Sherfy & Kirkpatrick, 1999), contributing to postburial persistence and viability (Mohamed‐Yasseen et al., 1994). Together, these seed traits along with tidally‐driven recurring sediment deposition can engender the formation of highly stratified seed banks that persist for decades to millennia (Brush, 2001; Jarrell et al., 2016; Lee, 1992; Peterson & Baldwin, 2004; Saunders, 2003; Törnqvist et al., 2004).

Profiles of *S*. *americanus* seed banks have proven to be a useful resource for a diverse range of research pursuits. For example, profiles have been used to reconstruct salinity conditions and sea‐level rise (Saunders, 2003; Törnqvist et al., 2004) because how much *S*. *americanus* primary productivity contributes to soil organic matter accumulation depends on salinity (Choi et al., 2001; Langley et al., 2013; Ross & Chabreck, 1972). Seed bank profiles have also been used to inform demographic shifts of *S*. *americanus* across historical time (Jarrell et al., 2016; Saunders, 2003). Notably, Summers et al. (2018) demonstrated that *S*. *americanus* seeds could be revived to reconstruct century‐long records of genetic variation and to assemble experimental populations to study eco‐evolutionary responses to environmental change. Blum et al. (2021) also reconstructed a century‐long record of evolution, focusing on the gain and loss of salinity tolerance in *S*. *americanus* relative to estuarine conditions in the Chesapeake Bay.

### 2.2 Seed collections

**FIGURE 1:**
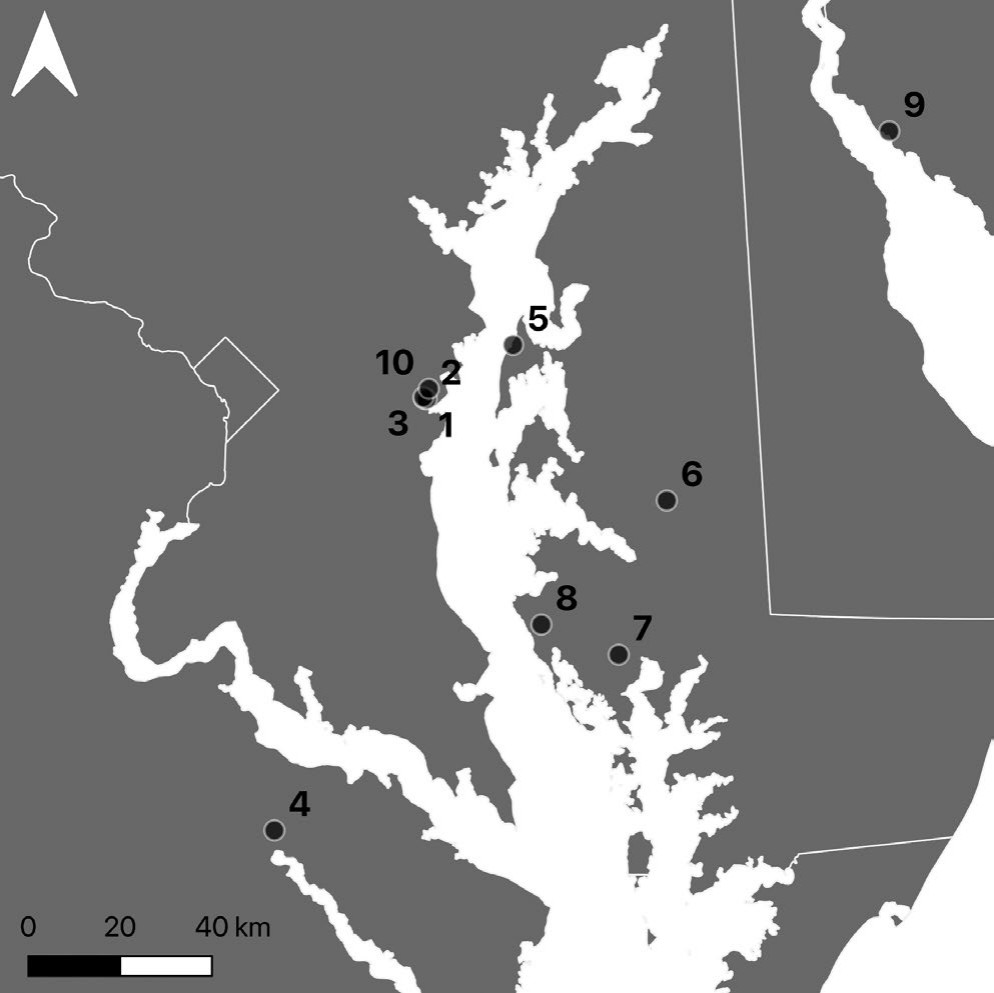
Seed source locations (i.e., provenance) in the Chesapeake Bay region of the mid‐Atlantic coast of the United States. 1 = Kirkpatrick Marsh, 2 = Corn Island, 3 = Hog Island, 4 = Virginia, 5 = Bay Bridge, 6 = Eastern Shore, 7 = Blackwater Wildlife Refuge, 8 = Taylors Island, 9 = Delaware Bay, 10 = Sellman Creek. Locations 1, 2, 3, and 10 are located at the Smithsonian Environmental Research Center and comprise 73.4% of all seeds used in germination trials

We obtained all soil‐stored seeds from marshes in the Chesapeake Bay and Delaware Bay watersheds (Figure 1, Table 2). Kirkpatrick Marsh was the source of 34.4% of all seeds (3644 of 10,588 seeds) used in germination trials. Kirkpatrick Marsh is a tidal brackish marsh on the Rhode River, a subestuary of the Chesapeake Bay in Maryland (USA) with a tidal range of 44 cm and a salinity range of 4–15 ppt (Keller et al., 2009). Since 1987, Kirkpatrick Marsh has hosted a CO_2_ enrichment study (Drake, 2014) and several other studies of marsh responses to environmental change (Langley & Megonigal, 2010; Langley et al., 2013; Noyce et al., 2019). We collected a monolith of soil (30 cm diameter, 35 cm deep) from Kirkpatrick Marsh in 2002 and a comparable monolith (30 cm diameter, 50 cm deep) from a study site on Delaware Bay in 2008. We also obtained seeds from this location and in three neighboring locations in the Rhode River basin (Figure 1, Table 2; locations 2, 3, and 10) in 2009 and 2017 and from six other locations across the Chesapeake Bay and Delaware Bay between 2008 and 2018 (Figure 1, Table 2; locations 4–9) with 25.4 cm diameter, 50–65 cm schedule‐30 PVC cylinder cores. For comparison, we also obtained contemporary seeds from accessions being grown in greenhouses originating from the Chesapeake Bay and Delaware Bay study sites (locations 4 & 9).

**TABLE 2:** Number of *Schoenoplectus americanus* seeds planted in germination trials collected from 10 salt marsh locations as well as from plants grown in a greenhouse (“Greenhouse”). Soil age (“Decade”) is rounded to the nearest decade and was estimated using the parameters in the statistical model that calibrated ^210^Pb profiles and soil depth in Kirkpatrick Marsh. The number of seeds germinated out of the number of seeds planted is shown in parentheses

Decade	Location										
	1	2	3	4	5	6	7	8	9	10	NA
	Kirkpatrick	Corn Island	Hog Island	Virginia	Bay Bridge	Eastern Shore	Blackwater	Taylors Island	Delaware Bay	Sellman Creek	Greenhouse
1770	–	–	1 (0)	–	–	–	–	–	–	–	–
1780	–	24 (0)	–	–	–	–	–	–	–	–	–
1790	–	3 (0)	–	–	–	–	–	–	–	2 (0)	–
1800	–	18 (0)	–	–	–	–	–	–	–	4 (0)	–
1810	2 (0)	28 (0)	–	–	–	–	–	–	–	2 (0)	–
1820	–	18 (0)	–	–	–	–	–	–	–	18 (0)	–
1830	1 (0)	39 (0)	–	–	–	–	–	–	–	17 (0)	–
1840	12 (0)	36 (2)	–	5 (0)	–	–	–	–	–	78 (3)	–
1850	1 (0)	17 (0)	5 (0)	–	–	–	–	–	–	25 (0)	–
1860	23 (0)	49 (0)	8 (0)	11 (0)	–	–	–	–	–	34 (0)	–
1870	330 (0)	128 (0)	8 (0)	–	–	–	–	–	–	2 (0)	–
1880	502 (1)	58 (0)	–	32 (0)	–	–	–	–	–	3 (0)	–
1890	356 (0)	157 (1)	13 (0)	–	–	–	–	–	–	–	–
1900	587 (11)	179 (8)	–	–	–	–	216 (1)	–	–	6 (0)	–
1910	132 (0)	248 (0)	–	11 (1)	–	–	144 (1)	–	–	13 (0)	–
1920	164 (2)	519 (15)	–	31 (2)	–	–	–	–	–	15 (0)	–
1930	48 (0)	159 (8)	3 (0)	33 (2)	2 (1)	–	144 (10)	1 (0)	–	4 (0)	–
1940	424 (10)	111 (6)	2 (0)	–	–	–	117 (3)	–	–	10 (1)	–
1950	261 (44)	156 (2)	9 (1)	41 (0)	–	–	60 (0)	–	–	38 (4)	–
1960	109 (41)	95 (2)	19 (1)	–	–	–	44 (0)	–	–	44 (3)	–
1970	257 (60)	134 (2)	29 (5)	12 (0)	–	–	258 (26)	2 (0)	2 (0)	74 (3)	–
1980	31 (4)	125 (2)	7 (2)	11 (4)	2 (1)	3 (1)	231 (42)	–	–	97 (10)	–
1990	141 (12)	125 (11)	27 (7)	14 (1)	–	8 (0)	270 (44)	2 (1)	21 (5)	116 (19)	–
2000	214 (65)	126 (7)	22 (6)	–	–	7 (0)	276 (116)	5 (0)	–	219 (50)	–
2010	–	144 (14)	37 (11)	–	–	–	419 (141)	–	–	98 (24)	62 (7)
2020	49 (13)	175 (44)	18 (7)	–	–	–	324 (118)	–	–	125 (9)	–
Total	3644	2871	208	201	4	18	2503	10	23	1044	62

We stratigraphically isolated seeds from the soil monoliths and cores. We first cut each core and monolith perpendicular to the vertical axis in 2 cm‐thick segments and then washed the segments over a 1 mm sieve. We visually identified and counted all *S*. *americanus* seeds remaining on the sieve and stored them in freshwater conditions at 4°C. We excluded cracked and partial seeds (i.e., empty seed coats) in subsequent germination trials.

### 2.3 Assessment of seed age and seed bank stratification

To verify the age and sedimentary stratification of seeds, we dated three sediment cores collected from Kirkpatrick Marsh according to radioisotope activity (Supplemental Materials). We relied on depths and age estimates of sediment from Kirkpatrick Marsh to approximate the ages of other seeds used in the study, understanding that the sediment accumulation rate likely varies among the sampled marshes depending on local hydrology, sediment loads, and tidal inundation patterns (Pethick, 1981). We accounted for some of this variability using information from the three sampled cores in the statistical modeling described below.

### 2.4 Germination experiments

We conducted 13 germination experiments from 2003 to 2019 using *S*. *americanus* seeds sieved from the soil monoliths and cores (Table S1; see Supplementary Materials for details). Each experiment was treated as an independent investigation, with the exception of a series of continuous germination trials with no clear start and end date that were conducted in 2016–2017, which we grouped as a single experiment. We note that data from the first two germination experiments were previously analyzed in Summers et al. (2018). We manipulated growing conditions such as temperature (four levels: 25°C, 30°C, 20°C daytime/15°C nighttime, 27°C daytime/15°C nighttime), media (three levels: sand, sand and soil mix, growth media [Murashige and Skoog salt and vitamin, sucrose, and agar mixture]), pretreatment of seeds (yes or no), and photoperiod (three levels: 15 h light/9 h dark, 12 h light/12 h dark, 0 h light/24 h dark) within and across the experiments, to identify optimal conditions for germinating seeds (Table S1, Supplemental Materials). While multiple pretreatments such as bleach and gibberellic acid were used to increase germination success, preliminary analysis suggested that there were no differences among pretreatments (Figure S2), so we pooled all pretreated seeds together to compare with those that were untreated.

The number of seeds used in each experiment varied considerably according to source (i.e., provenance) and soil depth (Table 2). For some source locations, we used only a subset of recovered seeds in germination trials, and some trials focused in particular on seeds recovered from deeper soil layers. All plants that germinated during the course of the experiments were transplanted into a 50:50 mixture of sand and potting soil (Fafard and Sons) and maintained in a greenhouse for later assessments of genetic and phenotypic variation.

### 2.5 Hierarchical models

We fit four hierarchical Bayesian regression models, each with a different statistical likelihood function, to data on germination success for all experiments combined. Germination success was defined as the number of successful germinants out of the number of seeds planted and was predicted by the fixed effects of seed age, experimental temperature, experimental medium, experimental photoperiod, and whether or not seeds were pretreated before the germination trial. We also included a random intercept as a grouping variable for seed provenance (11 locations). Thus, we quantified the proportion of seeds that germinated within each unique combination of experimental conditions, mean seed age (as predicted by seed depth; one of 75 possible depth intervals), and seed provenance. The proportion of seeds that germinate within a group of seeds of the same covariate values is synonymous with the probability that one seed within that group would germinate. We originally included a second random intercept to group observations by experimental assay, but this did not explain appreciable variation in germination success, so we removed the term from all subsequent models.

Using a hierarchical Bayesian approach allowed us to account for and explain important sources of variability in our data. First, we accounted for variability in estimates of the fixed‐effect, seed age. We calculated the age of groups of seeds of the same depth interval using a calibrated quadratic regression between soil depth and mean soil age estimated from ^210^Pb activity and bulk density (Figure S1, Supplemental Materials). We propagated variance across cores from that calibration through the hierarchical models using the following quadratic regression equation $x_{n}=\;\gamma_{0}\;+\;\gamma_{1}d_{n}+\gamma_{2}{d_{n}}^{2}+\;\epsilon_{n}$ where *d_n_* is the depth of the seed layer, *x_n_* is the predicted seed age in years, *γ_j_* (for *j* in 0, 1, 2) are regression coefficients, and *ϵ_n_* is residual error. Thus, the fixed effect of seed age for a group of seeds collected from the same depth from the marsh surface in the regression models is a random variable with a distribution (i.e., “errors‐in‐variables”; Dietze, 2017). We also accounted for variation in germination success by including a random intercept for seed provenance. Finally, we partitioned variability in model estimates of germination success by including fixed effects describing experimental conditions. Mixed effects modeling approaches like this (Bolker et al., 2009) can help overcome uneven representation across source locations and experimental treatments (Gelman et al., 2013), allowing for better determination of how each factor contributes to variability in germination success.

### 2.6 Model likelihoods and fitting

All of the hierarchical regressions had a binomial likelihood structure because our response variable was a proportion (i.e., seeds germinated/seeds planted) for groups of seeds that shared the same experimental treatment combination, seed provenance, and seed depth (i.e., predicted mean age; Table 1). Preliminary assessment of the germination data suggested that they could be zero‐inflated (ZI) and/or, more generally, overdispersed (OD) (Figure S3). Thus, to find the best fit model, we constructed four models with or without zero‐inflation and overdispersion components within a binomial regression. Accordingly, Model 1 had a generic binomial likelihood (−ZI/−OD), Model 2 had a zero‐inflated binomial likelihood (+ZI/‐OD), Model 3 had a beta‐binomial likelihood (−ZI/+OD), and Model 4 had a zero‐inflated beta‐binomial likelihood (+ZI/+OD) (Table 1; see Supplementary Materials for full model specifications).

These models can be linked to hypothesized biological mechanisms underlying germination success (Table 1). As zeros can arise from two separate processes in zero‐inflated models (Hooten & Hefley, 2019), we hypothesized that failed germinations due to seed inviability were related to the Bernoulli portion of the zero‐inflated model (excess zeros) and zeros resulting from failure to break the dormancy of viable seeds were related to the binomial portion of the zero‐inflated model. Specifically, we hypothesized that seed viability decreased with seed age as a separate process from the germination success of viable seeds declining with seed age while also being mediated by experimental conditions and seed provenance (Table 1). For the beta‐binomial models that accounted for overdispersion, we hypothesized that overdispersion could have arisen because we did not account for important covariates related to seed quality (e.g., seed size, thickness of seed coat) or the environment in which seeds were buried (Table 1).

We fit each hierarchical Bayesian model using STAN in the computing environment R (version 4.0.3; R Core Team, 2019), which is a program for Hamiltonian Monte‐Carlo Bayesian sampling (*rstan* version 2.21.1; Stan Development Team, 2020). We determined that running each model with three chains for 10,000 iterations (2000 warm‐up) with a thinning interval of three iterations allowed for convergence of all coefficient estimates. We used the following R packages for data manipulation, postprocessing, and plotting: *tidyverse* (version 1.3.0; Wickham et al., 2019), *ggmcmc* (version 1.4.1; Fernández‐i‐Marín, 2016), *loo* (version 2.3.1; Vehtari et al., 2020), and *cowplot* (version 1.0.0; Wilke, 2019).

### 2.7 Model checking and selection

We used posterior predictive checks (Gelman et al., 2014), one of the most common forms of model checking in Bayesian statistics (Conn et al., 2018), as the primary criteria for assessing the fit of the four competing hierarchical models. At each iteration of the Monte‐Carlo sampling procedure, we simulated a dataset of the same size and structure from the posterior distribution using only the model parameter values at that iteration. We then calculated three summary statistics of interest (mean number of seeds germinated across all groups of seeds, standard deviation of the number of seeds germinated across all groups of seeds, the number of groups of seeds with zero germinants) for each of the simulated datasets. This resulted in a distribution of summary statistic values (one value for each Monte‐Carlo iteration) for comparison with the summary statistic derived from the experimental data. We inferred that a candidate model could reasonably give rise to our data if the observed values fell within the 95% quantiles of the summary statistic distributions. We used Watanabe‐Akaike Information Criterion (Gelman et al., 2014; Watanabe, 2010) and LOO (leave‐one‐out) cross‐validation (Vehtari et al., 2017) as additional criteria for selecting the best fit model.

### 2.8 Seed viability tests

We used data from two tetrazolium seed viability assays (Lacroix & Mosher, 1995) to further evaluate the hypothesis that two processes drive variability in germination trial data with seed age: (1) germination success declines with seed age because seeds decline in viability, and (2) germination success declines with seed age due to a greater difficulty in breaking seed dormancy. We conducted tests on a subset of seeds that failed to germinate (*n* = 470 seeds). We assessed whether the proportion of viable seeds that failed to germinate varied according to seed age using a binomial regression with seed depth as a fixed effect.

## 3 RESULTS

### 3.1 Seed age and stratigraphy

Sediments from Kirkpatrick Marsh, the origin of 34.4% of seeds for our germination trials, did not exhibit evidence of stratigraphic mixing according to radiometric analysis. A combination of ^210^Pb and ^137^Cs activity provided the approximate ages of the top 30 cm of sediment, encompassing nearly all seeds recovered from the site. Uncertainty in dating across the three cores was accounted for in the hierarchical Bayesian models by including seed age as a random variable (Figure S1). For example, a seed collected at a depth of 20 cm would have a 95% probability of being within 94 and 120 years old, with an estimated mean seed age of 107 years.

### 3.2 Model selection results

**FIGURE 2:**
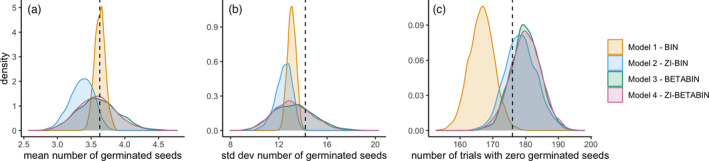
Comparisons of the four hierarchical models fit to germination trial data. Colored distributions are posterior predictive distributions for three summary statistics (a) mean number of seeds germinated, (b) standard deviation of the number of seeds germinated, and (c) number of trials with no successful germination. Black dashed lines in each of the panels represent the true value of the summary statistic from the observed data. Distributions that encompass the true value of the summary statistic suggest that the model could give rise to the observed data

Posterior predictive checks indicated that the best fit models were Model 3 (beta‐binomial) and Model 4 (zero‐inflated beta‐binomial) (Figure 2). Assessing posterior predictive checks for multiple summary statistics revealed why Models 1 and 2 did not fit the data as well as the other two models. Model 1 (binomial) was able to capture the true mean with high precision (Figure 2a), but it failed to capture the number of zeros (Figure 2c) and the spread in the data (Figure 2b). Model 2 (zero‐inflated binomial) successfully captured the number of zeros (Figure 2c) but underpredicted the mean (Figure 2a) and failed to capture the spread in the data (Figure 2b).

**FIGURE 3:**
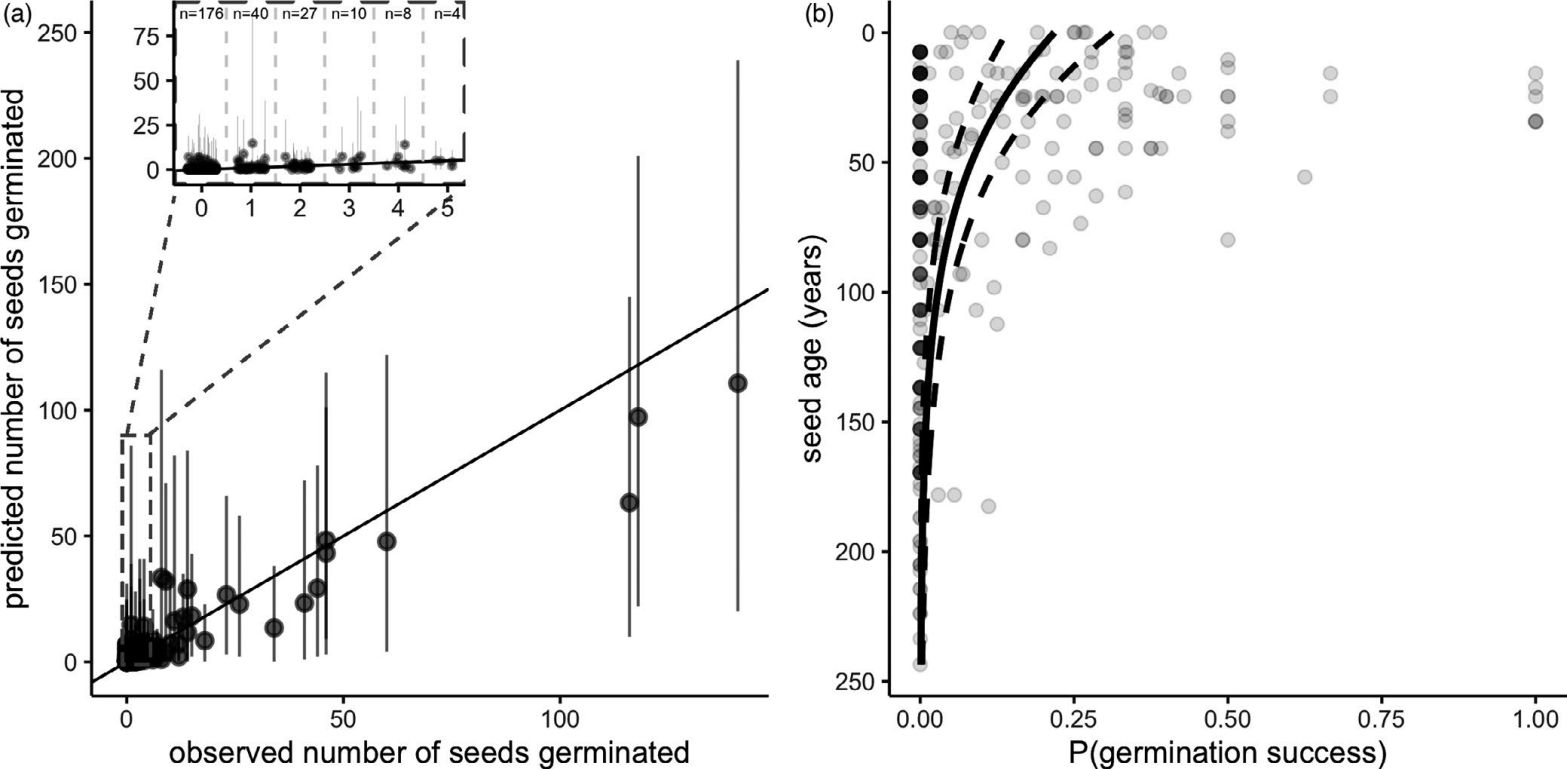
(a) Observed vs. predicted germination success from posterior predictive distributions of the beta‐binomial model without zero‐inflation (Model 3, *R* ^2^ = 0.87). Points represent a unique germination trial across seed age, seed provenance, temperature, media, pretreatment, and photoperiod (*n* = 298). Bars represent 95% credible intervals around predicted means. The inset graph highlights the high density of points where there were five or fewer germinants in a trial. (b) Predicted probability of germination from the beta‐binomial model without zero‐inflation (Model 3), averaged (with weighting) across germination trial conditions. The solid line represents the median, and the dashed lines represent the 95% credible interval. Raw data are depicted as points. Overlapping points with the same value (e.g., P(germination success) = 0) are shaded darker

Our model selection criteria corroborated our results from the posterior predictive model checks (Table S2). Models 3 and 4 were a better fit to our data than Models 1 and 2, whereas there was no appreciable difference between the fits of Models 3 and 4. Observed vs. predicted plots of the number of seeds germinated for each unique trial also indicated adequate model fit for Model 3 (Figure 3a, *R* ^2^ = 0.87) and Model 4 (Figure S4, *R* ^2^ = 0.87), while Models 1 and 2 overconfidently predicted germination probabilities (Figure S4). There was no evidence that the zero‐inflation component of the zero‐inflated beta‐binomial model (Model 4) varied with seed age (95% credible interval [CI] slope: [−10.50, 7.82]), and there were minimal differences in the predictive ability between Models 3 and 4 (Figure 2, Table S2). Therefore, we selected the beta‐binomial model (Model 3) as the best fit and most parsimonious model for subsequent analyses.

### 3.3 Predictors of germination success

Seed germination success declined exponentially with seed age in the beta‐binomial model (slope: −1.32, 95% CI [−1.62, −1.04], Figure 3b). On average, modern seeds were predicted to have a germination probability of 21.8% [14.1, 31.3], whereas seeds collected at 20 cm depths (estimated age: 107 years) were predicted to have a germination probability of 2.5% [1.3, 4.3].

**FIGURE 4:**
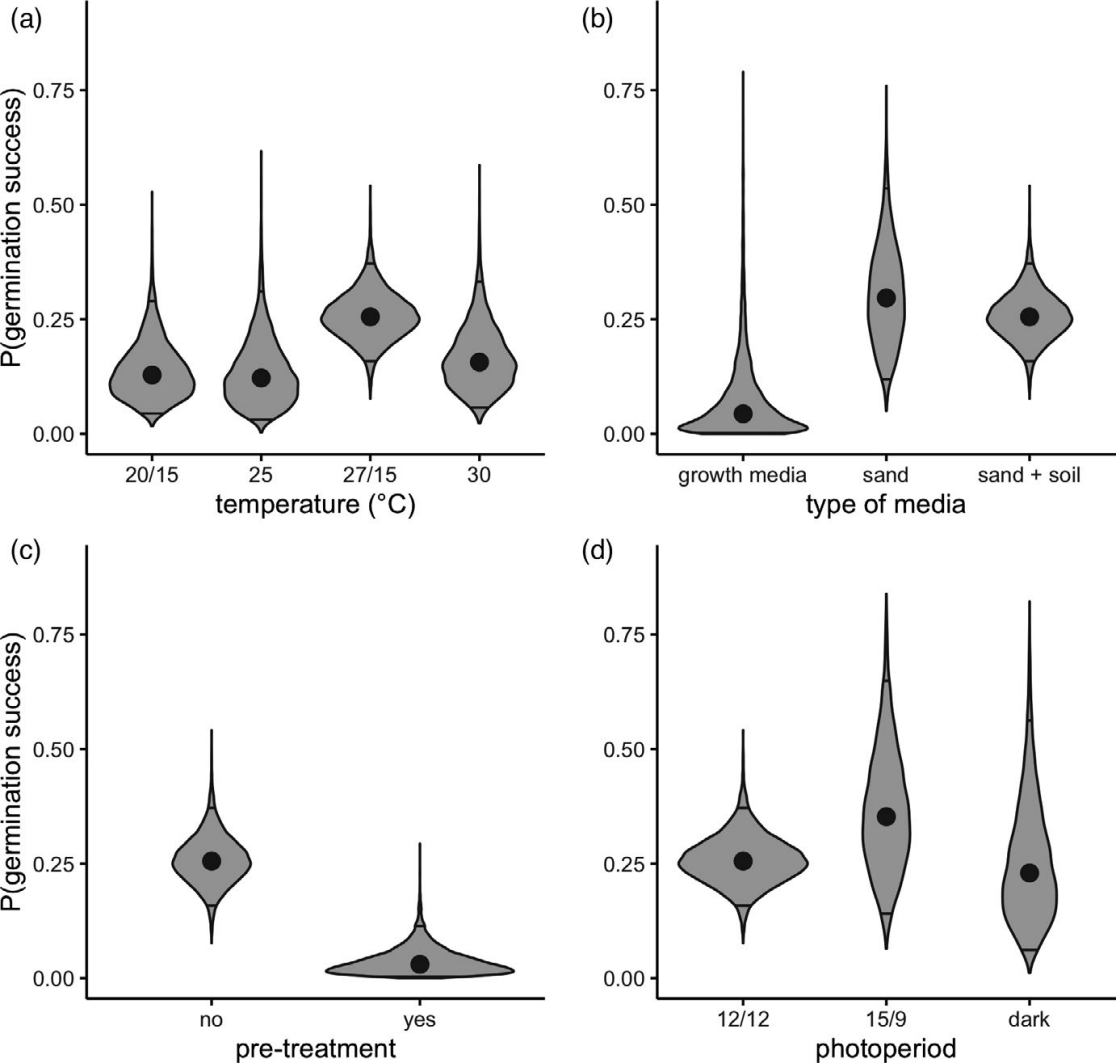
Predicted germination success of modern seeds for (a) temperature treatments, (b) media types, (c) whether or not seeds were pretreated, and (d) experimental photoperiod at reference level germination trial conditions from Model 3 (temperature = 27/15°, media = sand/soil, pretreatment = none, photoperiod = 12 h daytime/12 h nighttime). Shaded distributions are calculated from the marginal posterior parameter distributions. The median of each distribution is denoted with a point, and the 95% quantiles are shown as horizontal lines

Experimental conditions mediated the proportion of seeds that germinated in a trial (Figure 4). The choice of media and pretreatment of seeds were particularly influential in explaining average germination success. For example, for seeds collected near the soil surface, planting on sand and holding all other experimental conditions constant resulted in an average predicted germination probability of 29.7% [12.1, 53.4]. In contrast, those for which seed endosperm was grown on a growth medium (Murashige and Skoog salt and vitamin, sucrose, and agar mix) had a predicted germination probability of 4.4% [0.1, 31.6] (Figure 4b). Seeds near the marsh surface that were pretreated (e.g., bleach, gibberellic acid) had an average predicted germination probability of 3.0% [0.4, 11.4] while untreated seeds had an average predicted germination probability of 25.5% [16.0, 37.1] (Figure 4c). Temperature and photoperiod had a lesser influence on germination rates on average than did the media and pretreatment the seeds experienced. However, warmer, fluctuating temperatures (Figure 4a), and having a 15‐h daytime/9‐h nighttime photoperiod (Figure 4d) promoted germination.

**FIGURE 5:**
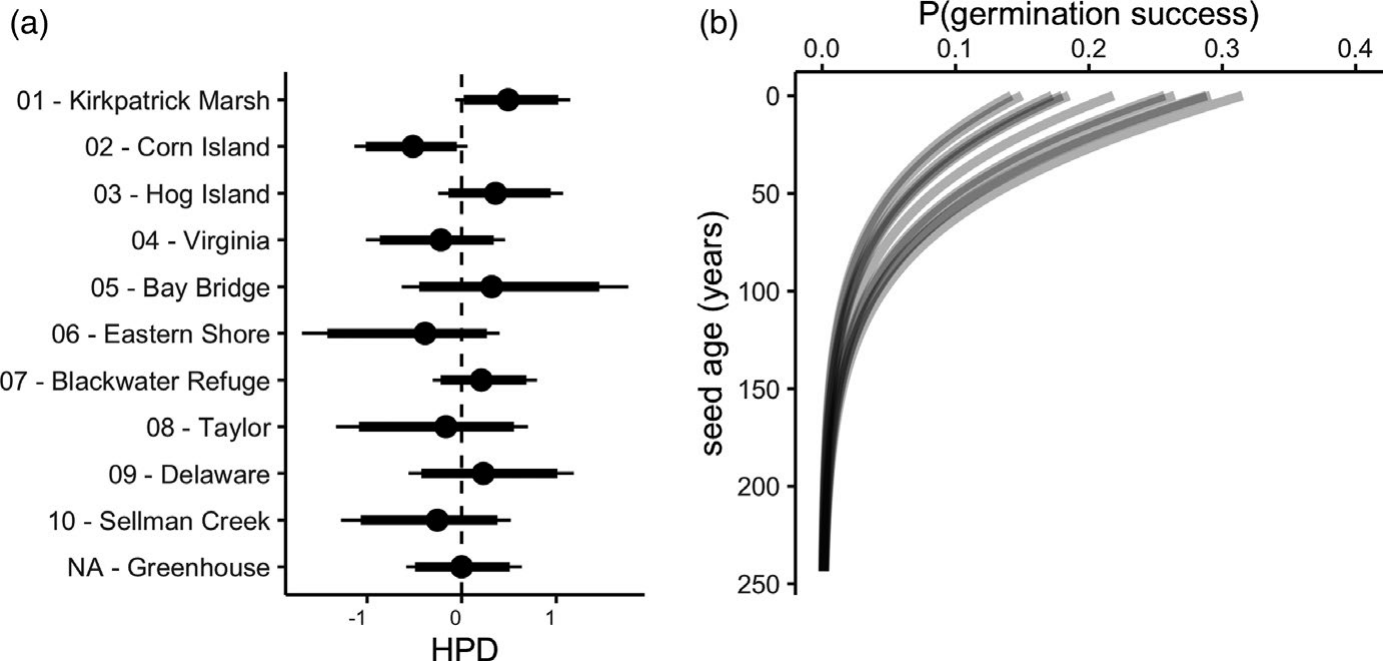
The effect of seed provenance on germination success from the beta‐binomial model without zero‐inflation (Model 3). Points in (a) represent predicted mean values of random effect deviations from the global mean with thick and thin bars representing 90% and 95% quantiles of the highest posterior density (HPD), respectively. Mean predicted germination success across seed age is shown for each location (b) for ease of interpretation. “Greenhouse” represents seeds collected from plant accessions grown in a greenhouse

Seed provenance explained considerable variation in germination success (Figure 5). The largest difference attributable to seed provenance occurred between Kirkpatrick Marsh and Corn Island, which are both located at the Smithsonian Environmental Research Center (Figure 1, locations 1 & 2). The difference in average predicted germination probability between these locations for seeds at the shallowest depths (difference = 17.3% [12.5, 21.6]) was comparable in magnitude to differences attributable to experimental conditions in the germination trials.

### 3.4 Seed viability tests

**FIGURE 6:**
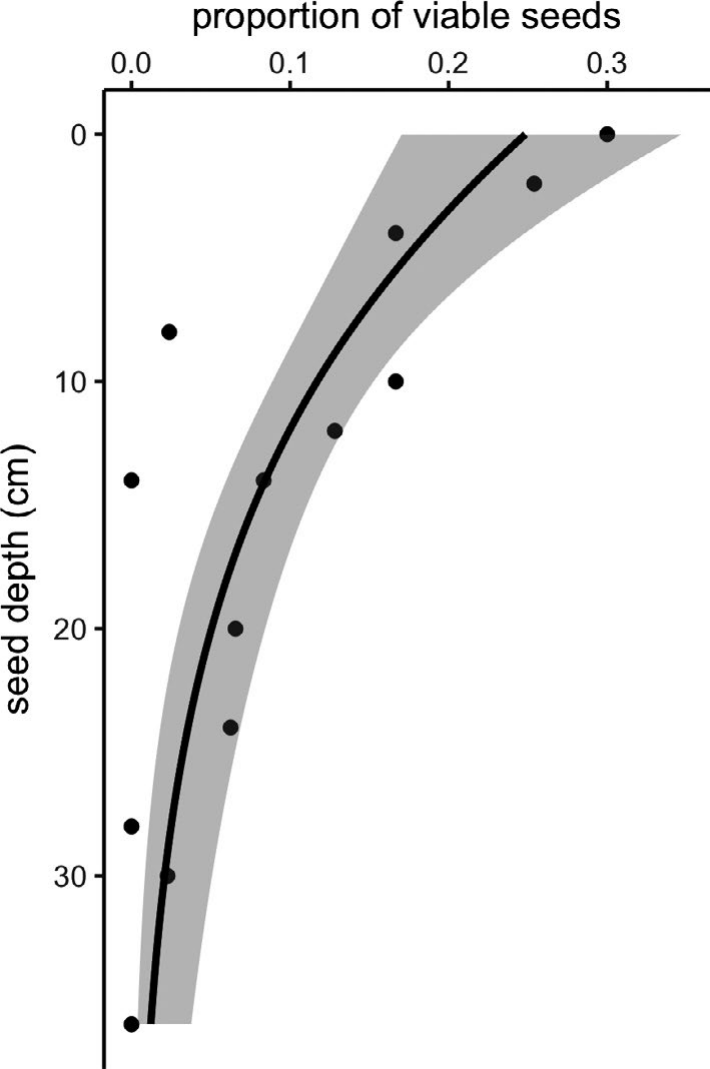
Proportion of viable seeds by seed depth according to tetrazolium tests of seeds that failed to germinate in germination trials. Dots represent an independent tetrazolium trial and depth. The bolded line represents the predicted mean from a binomial regression, and shaded areas represent 95% confidence intervals

Most seeds that failed to germinate in the subset of trials for which we conducted tetrazolium tests were inviable: only 10.4% of seeds tested were determined to be viable using tetrazolium as an indicator. The proportion of tetrazolium‐determined viable seeds decreased with seed depth (Figure 6). This suggests that declines in germination success are more likely driven by declines in seed viability than limitations of the methods used to germinate seeds and corroborates that a zero‐inflated component is not necessary to effectively model the distribution of the observed data.

## 4 DISCUSSION

Understanding sources of variability and bias in propagule viability can strengthen inferences drawn from soil‐stored natural archives about biotic responses to environmental change. We conducted a hierarchical statistical analysis using data from 13 germination experiments accounting for and explaining variability in *S*. *americanus* germination success, a proxy for seed viability. Using a model selection approach, we found that a beta‐binomial model best fit our experimental data, indicating our data were overdispersed, but not zero‐inflated. We corroborated results from our model selection analysis with seed viability data from tetrazolium tests, indicating that seed viability declined with seed age more so because of progressively lower viability than decreasing ability to break the dormancy of still viable seeds. Our model also revealed that germination success varied by methods to break seed dormancy and the provenance of the seeds. Our findings illustrate how gaining insight into variability around the persistence and viability of soil‐stored seeds can help ameliorate some concerns about latent bias and thus help guide the assembly of experimental cohorts to reconstruct decadal to century‐long records of the evolutionary responses of plants to environmental change.

### 4.1 Intrinsic mechanisms mediating germination success

To assess how seed germination success varied with seed age, we explicitly linked our statistical hypotheses (i.e., choice of model likelihoods) with our understanding of seed banking ecology. We proposed that variability in our germination trial data could be explained by two separate processes that result in seed germination failure and/or our failure to measure important characteristics of the seeds. We then proposed statistical models that would best capture these potential sources of variability in the data. While our germination trial data did exhibit a large number of zeros (Figure S3)—with 59.1% of observations having zero germinants—a zero‐inflated component was not necessary nor adequate in capturing variation in germination success (Figure 2). This result corroborates the findings of other ecological modeling analyses that explicitly compare zero‐inflated (e.g., zero‐inflated Poisson) and overdispersed models (e.g., negative binomial) for count data (Sileshi, 2008; Vaudor et al., 2011). Given this phenomenon, comparing the performance of zero‐inflated and overdispersed models when fitting data with a large number of zeros is recommended (Warton, 2005; Warton et al., 2016) as we did here because these distributional assumptions can significantly impact downstream statistical inference (Vaudor et al., 2011).

### 4.2 Seed viability and viability decay

Consistent with extrapolations suggesting that sedge seeds can persist for 15 to ≥130 years (Schütz, 2000) we found that *S*. *americanus* seeds can remain viable for more than a century (Figure 3b). Seeds that endure for more than three to five years are generally characterized as persistent (Thompson et al., 1998), suggesting that soil‐stored *S*. *americanus* seeds exhibit remarkable resilience to aging and environmental exposure. In contrast to some celebrated examples of exceptional longevity, such as *Acacia* and *Lotus* seeds, indicating that dry storage conditions promote persistence for centuries to millennia (Daws et al., 2007; Leino & Edqvist, 2010; Long et al., 2015; Shen‐Miller et al., 1995), our findings affirm that burial due to recurring deposition of sediment and plant detritus combined with soil saturation can promote *in situ* persistence of seeds (Bennington et al., 1991; Fennell et al., 2014; McGraw et al., 1991; Morris et al., 2002; Vavrek et al., 1991). This likely occurs because burial and inundation result in low, stable temperatures and anoxic conditions that reduce microbial‐driven decomposition (Lee, 1992; Probert et al., 2009). Our results also support prior work showing that traits such as a small, nearly spherical size and a durable coat, characteristic of *S*. *americanus* seeds, can contribute to seed persistence in soil (Bakker et al., 1996; Bass, 1980; Fox, 1983; Honda, 2008; Mohamed‐Yasseen et al., 1994; Moody‐Weis & Alexander, 2007; Schwienbacher et al., 2010). Consideration of both factors raises the possibility that highly persistent soil‐stored seed banks are much more widespread than is currently thought, as sedges and other plants with seeds exhibiting traits that engender persistence often dominate ecosystems with wet, anoxic soils such as tundra, heathlands, glades, marshes, and mangroves that collectively have a large global footprint.

Though *S*. *americanus* seeds remain viable for a century or more, we found that germination rates declined with increasing age (i.e., depth). Germination probability declined from an average of 22% in recent sediments to 3% in century‐old sediments (Figure 3b). The estimated rate of decline in the germination of soil‐stored *S*. *americanus* seeds falls within the range of rates estimated for decades‐ to century‐old seeds in museum and herbarium collections. For instance, germination rates of seeds in the Museum of Natural History in Paris varied from 0% after 55 years (*Melilotus lutea*) of storage to 100% after 158 years (*Cassia multijuga*) of storage (Becquerel, 1934; Bewley et al., 2013). The estimated rate of decline also falls within the range of rates inferred from long‐term burial experiments. Rates estimated from the Beal’s buried‐seed experiment started in 1879 vary considerably, with average annual rates ranging from 2.5% with ≤40‐year longevity (*Capsella bursa*‐*pastoris*) to 0.9% (*Oenothera biennis*) and 0.18% (*Verbascum blattaria*) with ≥120‐year longevity (Kilivaan & Bandurski, 1981; Telewski & Zeevaart, 2002). The estimated rate of decline in the germination of soil‐stored *S*. *americanus* seeds is comparable or greater than declines estimated for other soil‐stored dormant propagules. For example, some *Daphnia* ephippia exhibit up to 75% revival over century‐long sedimentary records (Burge et al., 2018; Frisch et al., 2014; Hairston et al., 1995; Weider et al., 1997). However, we suggest that germination rates found in this study can provide ample sampling of historical cohorts for eco‐evolutionary studies (Blum et al., 2021; Summers et al., 2018), particularly at more recent seed ages, with the caveat that seed traits are not strongly genetically correlated with adult plant traits. Future empirical work is warranted to assess the strength of bias due to correlations between seed and plant traits and between traits related to dormancy and plant traits as emphasized by Weis (2018).

Testing the fit of a zero‐inflated model to our data allowed us to assess the hypothesis that decline in seed germination success with seed age could arise from two processes: increases in the likelihood of seeds being inviable with age or declines in our ability to break the dormancy of older seeds that have been buried in sediment for longer. Both processes could result from progressive deterioration of seeds due to microbial degradation or more prolonged exposure to unfavorable environmental conditions. It is also possible that a decline in germination might reflect temporal shifts in genetic variation, as has been observed in *S*. *americanus* (Summers et al., 2018) because traits related to persistence or dormancy can be heritable (Foley & Fennimore, 1998). Our model selection analysis showed that a zero‐inflated model did not adequately capture the variation in our experimental data (Model 2) and did not contribute to inferential power when added to the beta‐binomial model (Model 4). This suggests that separating intrinsic (i.e., declining viability) and operational (i.e., inability to break dormancy) factors that can result in germination failure was not necessary to explain the variability in the germination of *S*. *americanus* seeds given the data from our germination trials.

Empirical assays of seed viability support our model‐based inferences. Tetrazolium tests supported the inference that seed viability declines with seed age: there was no evidence to suggest that the decline in germination with seed age found in our trials was due to an increasing inability to break the dormancy of viable seeds (Figure 6). This is a promising result as it suggests nondestructive germination trial data are an adequate proxy for destructive tetrazolium viability testing. Thus, assessing seed viability and using resurrected seeds for eco‐evolutionary experiments need not be separate endeavors.

While our results indicate that statistical separation of zero‐generating ecological processes was not imperative to understanding how seed germination success declines with seed age for our experiments, it is nonetheless important to recognize that different phenomena can influence seed germination success and viability and that there were indeed likely seeds that failed to germinate because they were inviable and those that failed to germinate because of our inability to break their dormancy. A zero‐inflated modeling framework might still prove useful for estimating viability for particular age cohorts or other hatching and germination experiments, which tend to generate data with zeros that can reflect different underlying processes. Within the statistical literature, zero values are referred to as true and false zeros (Hooten & Hefley, 2019), structural zeros (Warton et al., 2016), or excess zeros. Regardless of how many zeros are observed in the data and the inclination to separate zeros in a statistical framework, we concur with broader recommendations (e.g., Warton, 2005; Hooten & Hefley, 2019) that the choice to do so should be motivated first by how well a model fits the data according to similar model checking and selection approaches illustrated in this study.

### 4.3 Predictors of germination success

The results from our best fit model indicate that germination success varied by experimental conditions (Figure 4), with the largest differences in germination success arising from the media on which seeds were germinated (Figure 4b) and whether or not seeds were pretreated (Figure 4c). Temperature regime also mediated germination success; in particular, germination success was higher when seeds were exposed to warmer daytime temperatures (Figure 4a). Similarly, there was some indication that germination success was optimized under a fluctuating temperature regime (Figure 4a). This is consistent with prior work showing that the ability to break the dormancy of sedge seeds increases with temperature (Kettenring & Galatowitsch, 2007), and that a 10–12°C temperature fluctuation is an optimal treatment for germinating seeds from several species (Dietert & Shontz, 1978; Kettenring & Galatowitsch, 2007; Wagner & Oplinger, 2017a, 2017b). We found that photoperiod had minimal influence on germination success, which corroborates findings from germination trials of ecologically similar sedge, rush, and grass species (Wagner & Oplinger, 2017a, 2017b). Notably, after controlling for temperature, media, pretreatment, and photoperiod there were no discernable differences in germination success across experiments, as indicated by a random effect variance near zero for grouping by experimental assay (results not shown). This suggests that other unmanipulated experimental conditions did not contribute substantially to variation in germination success in this study. While our findings offer some insight about the merits of experimental optimization, additional experiments explicitly designed to identify optimal germination conditions are warranted to increase understanding of what best breaks the dormancy of highly persistent, soil‐stored seeds of *S*. *americanus* (Marty & Kettenring, 2017).

We found that seed provenance accounted for a considerable amount of the observed variation in germination success (Figure 5). This is consistent with prior work showing that the persistence of seed banks can be geographically variable (Leck & Schütz, 2005) and that variation in germination of marsh sedge seeds can be strongly influenced by their geographic source (Marty & Kettenring, 2017). It also parallels evidence that hatching rates of dormant *Daphnia* ephippia vary according to provenance (Radzikowski et al., 2018). Variation due to provenance may result from differences in long‐term exposure to environmental conditions that influence propagule persistence and viability. For example, hatching rates of *Daphnia* ephippia can be depressed by long‐term exposure to heavy metals in sediments (Rogalski, 2015). In coastal marsh environments, persistence might reflect local hydrology such as tidal regime, nutrient inputs, and other factors such as temperature that can moderate decomposition (Baskin & Baskin, 1998).

Interestingly, the most extreme differences in germination success across locations were between Kirkpatrick Marsh and Corn Island, two geographically proximate sites at the Smithsonian Environmental Research Center in the Chesapeake Bay (Figure 1, locations 1 and 2). Observed differences between the two sites could reflect fine‐scale intraspecific genetic differentiation. Prior work has shown that *S*. *americanus* exhibits genetic differentiation within and among marshes (Blum et al., 2010; Summers et al., 2018). Germination rates can be moderately to highly heritable (e.g., Saeidi, 2008), and like other life history attributes (Reznick et al., 1997), the extent of heritability might differ among genetically distinct (sub)populations of *S*. *americanus*. This hypothesis is supported by evidence that seed persistence can vary among populations (Kochanek et al., 2009) and evidence that hatching rates of *Daphnia* ephippia vary by familial descent (De Meester & De Jager, 1993). Additional assays are thus needed to better understand how spatially variable extrinsic and intrinsic factors contribute to germination variability.

It is important to note that our inferences are constrained by the number of dated sediment cores used to inform our estimates of seed age, with all three dated cores collected from Kirkpatrick Marsh (Figure 1; location 1). Thus, it is possible that variation in germination success explained by seed provenance could have arisen from differences in the relationship between seed depth and seed age across locations rather than the proposed alternatives above. It is reasonable to expect that sedimentation rates and other relevant biogeochemical processes governing sedimentation and seed age by depth vary, particularly for geographically disparate locations (e.g., sites in Chesapeake Bay vs. sites in Delaware Bay). Although we sought to incorporate some of this variation in our estimates of seed age using variability across sediment cores (Figure S1), future analyses should more explicitly account for this concern to disentangle differences in seed viability due to seed age and differences due to other biogeochemical characteristics of the source location(s) (i.e., provenance).

### 4.4 Future work

Here, we identified and accounted for some biases and sources of variation that arise when using germination data of soil‐stored seeds to serve as a proxy for seed viability, including experimental conditions and distinguishing between viable and inviable seeds as a function of seed age. While we do not address all significant biases of using resurrected propagules in eco‐evolutionary studies (e.g., “the invisible fraction”; Weis, 2018), we do provide a framework for integrating data and statistical models that could be useful in future studies. Importantly, our approach emphasizes accounting for uncertainty using hierarchical Bayesian models, which can be useful when data are limited (McNeish, 2016) or when ecological processes are nonlinear (Hobbs & Hooten, 2015). We contend that accounting for uncertainty will continue to be important in conceptualizing how plant populations have evolved over historical time, a process that will likely always be hindered by a data limitation problem (Franks et al., 2018), but wherein some data are better than none at all in attempts to reconstruct ecosystem structure and function of the past. While challenges within the field are often focused on the possibility of biased representation of sampled resurrected propagules to their historical cohort (e.g., Bennington & McGraw, 1995), a nuanced modeling approach could also account for the unbiased sampling error that arises due to small sample sizes.

## 5 CONCLUSIONS

Our findings build on prior work (Blum et al., 2021; Jarrell et al., 2016; Saunders, 2003; Summers et al., 2018), indicating that *S*. *americanus* can serve as a model for studying persistent soil‐stored seed banks and for using dormant propagules to infer evolutionary change of an ecosystem engineer over ecologically‐relevant timescales. Evidence that *in situ* viability of *S*. *americanus* seeds extends for a century or more helps lay the foundation for further inquiry about the ecophysiology, environmental conditions, and evolutionary drivers of aging, decay, and dormancy of soil‐stored seeds (Long et al., 2015). Using a hierarchical Bayesian modeling approach, we accounted for and gained valuable perspective on what underlies variation in germination data using seeds resurrected from soil‐stored seed banks. By complementing other recent findings, such as evidence that genetic diversity of *S*. *americanus* plants revived from seeds does not decline with time since burial (Summers et al., 2018), insights gained from our study offer further support for the premise that persistent and stratified soil‐stored seed banks can serve as resources for reconstructing decadal to century‐long records of plant responses to environmental change. Importantly, we show that declines in germination success with age are more likely due to declines in seed viability rather than increasing failure to break dormancy, indicating that germination trial data are likely an adequate proxy for seed viability. However, given that our germination data were overdispersed, we suggest that further advances could come by accounting for seed traits such as coat thickness or seed size, and data on the characteristics of the sediment in which seeds were buried to explain variation in germination success and viability better.

Our work also offers some guidance for breaking dormancy to assemble depth/age cohorts of *S*. *americanus* for time‐shift experiments (Blanquart & Gandon, 2013) to explore the role of adaptation in response to past and near‐term future environmental change (Bustos‐Segura et al., 2014; Davis et al., 2005; Orsini et al., 2013). With further refinement, the use of soil‐stored seed banks could provide more realistic contexts, in contrast to space‐for‐time approaches (Shaw & Etterson, 2012), for inferring the progression of evolution in natural populations (Blum et al., 2021), and thus eventually emerge as a powerful complement to similarly‐minded approaches that rely on *ex situ* seed archives (Etterson et al., 2016; Everingham et al., 2021; Franks et al., 2008; Summers et al., 2018; Weis, 2018).

## CONFLICT OF INTEREST

The authors declare no conflict of interest.

## Supporting information

Supplemental Materials

## Data Availability

All data and code are available via the Github repository (https://github.com/mlvahsen/SchoenoplectusGermination).
