# Supplementary material for: Accounting for variability when resurrecting dormant propagules substantiates their use in eco‐evolutionary studies
Source: Evol Appl. 2021 Nov 27;14(12):2831–47. doi: 10.1111/eva.13316 (PMC8674891; doi:10.1111/eva.13316)
Supplement: Supplementary file 1 — Supplemental Materials [file EVA-14-2831-s001.pdf]

# 1 Sediment Dating

Three cores were sampled in 2000 (two cores) and 2004 (one core) from Kirkpatrick Marsh at the Global Change Research Wetland within the Smithsonian Environmental Research Center property (Edgewater, Marlyand, USA) to estimate soil dates with depth below the marsh surface using radiometric radiometric analysis of $^{210}$Pb and $^{137}$Cs. Because accretion rates in Chesapeake Bay marshes have varied over the past 200 years [7, 6], we calculated soil dates using the constant rate of supply (CRS) model [2] that allows for variable accretion over time (see Supplemental Data for age estimates). The depth of peak $^{137}$Cs activity was also used as an independent marker of the depth corresponding to 1964. Sediment mixing might result in incorrect determination of seed ages [5]. We found that $^{210}$Pb activity in the three cores followed the same patterns: in the upper 10 cm of sediment root ingrowth may have occurred. Mudd *et al.* (2009) showed that this pattern could be attributed to living roots in the sediment at that depth [12]. This pattern was consistent across all three cores.

To account for uncertainty in predicted seed age across the cores, we fit a quadratic regression of sediment year predicted by sediment depth using all three cores. This calibration model was specified as:

$$
\begin{aligned}
y_i &\sim \text{normal}(\mu_i, \sigma_p^2) \\
\mu_i &= \gamma_0 + \gamma_1 d_i + \gamma_2 d_i^2 \\
\boldsymbol{\gamma} &\sim \text{multivariate normal}(\mathbf{0}, \mathbf{I}) \\
\sigma_p &\sim \text{gamma}(0.01, 0.01),
\end{aligned}
$$

where $d_i$ is depth of soil layer (cm) and $y_i$ is the predicted soil age (year of piston core sampling [2000 or 2004] – predicted soil year). We fit this model in *brms* [3] with 5000 iterations and 1000 burn-in iterations. The marginal posterior distributions:

$$
\begin{aligned}
\boldsymbol{\gamma} &\sim \text{multivariate normal}\left(\begin{bmatrix} -0.590 \\ 3.64 \\ 0.09 \end{bmatrix}, \begin{bmatrix} 6.76 & -0.98 & 0.03 \\ -0.98 & 0.21 & -0.01 \\ 0.03 & -0.01 & 2.79e^{-4} \end{bmatrix}\right) \\
\sigma_p &\sim \text{gamma}(32.55, 5.02),
\end{aligned}
$$

were used as informative priors in the four hierarchical seed germination models. We fit two alternative calibration models allowing for random intercepts and slopes grouped by individual core, but the simplest model (Fig S1) was the most parsimonious.

In lieu of better measurements of sediment age in the other marsh locations, we used estimates of the age of marsh sediments in Kirkpatrick Marsh to approximate the age of seeds from the other locations. Seed ages from these cores should therefore be considered with caution.

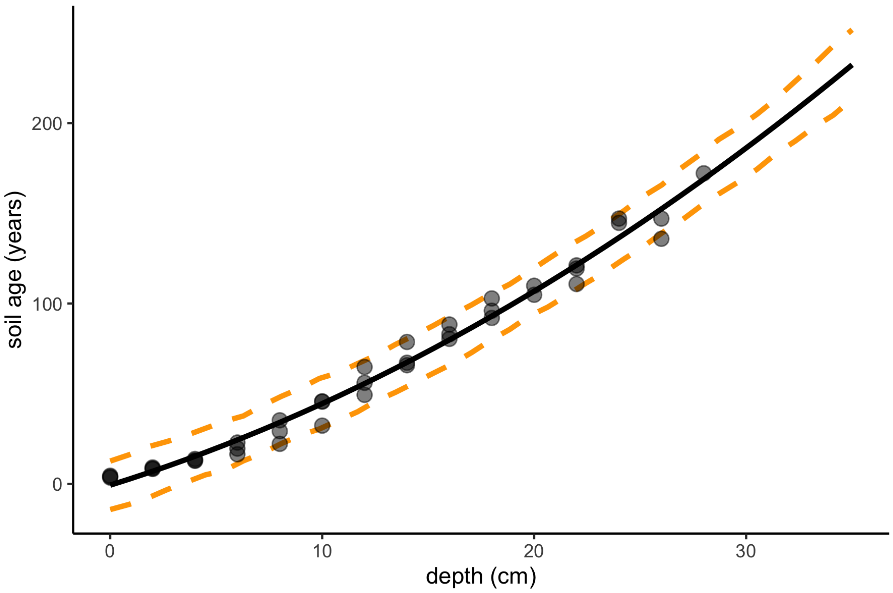


Figure S1: Predicted relationship between soil depth and age from the calibration model fit to $^{210}$Pb and $^{137}$Cs data from three piston cores. Raw data are shown as black circles, the predicted mean is shown as the black solid line, and the 95% prediction intervals are shown with the orange, dashed lines.

# 2 Experimental Assays

## Experiments 1 and 2

All seeds were planted in a 1:2 sterile sand and Ferry & Morse Seed Starter Mix (Ferry & Morse, Fulton, Kentucky, USA). Seeds were placed in the soil mixture, and the water level in a tray containing the germination pots was kept at approximately 1 cm below the soil surface. The first six days had a photoperiod of 24 hour light: 0 hr dark because of a mechanical problem. Afterwards, a 15 hour light: 9 hour dark photoperiod was maintained. Given that the majority of the time, seeds were under the 15/9 photoperiod, germination data from this trial was assigned to the 15/9 photoperiod grouping for statistical analyses. The temperature was kept at a constant 30°C. In May and June 2004, a second germination assay with remaining seeds was conducted following the same methods, except that a 15 hour light: 9 hour dark photoperiod was maintained for the duration of the study. Data from these experiments have been previously published in Summers *et al.* (2018) [16].

## Experiment 3

In April 2010, seeds were subjected to a variety of pretreatments (see Table S1) to break dormancy before being planted in a 1:2 mix of sterile sand and potting soil (Fafard and Sons, Milford, Massachusetts, USA). The pretreatments included bleach (130°F dH2O for 30 minutes, then soaked in sodium hypochlorite for 5 minutes, and rinsed with dH2O), water

(130°F dH2O for 30 minutes), bleach and water (8% sodium hypochlorite for 5 min then rinsed with dH2O), auxin ($1X10^{-3}$ M IAA dissolved in ethanol before dissolved in distilled water (50 mg/ml of ethanol) - seeds soaked for 24 hours for absorption [4]), ethephon (0.001 M dissolved in 100 mg/ml dH2O - seeds soaked for 10 minutes[15]), gibberellic acid (2.89 X 10-4 M GA dissolved in ethanol before dissolved in distilled water (200 mg/4 ml of water) - seeds soaked for 24 hours for absorption [13]), and no pre-treatment. Water level was maintained at 1 cm below the soil surface for two months. Temperature was kept at a constant 30°C and the photoperiod was 15 hours light: 9 hours dark.

We assessed differences in the effects of individual pre-treatments on germination success with this trial before incorporating pre-treatment as a fixed effect in the hierarchical model. We fit a binomial regression to the germination data from this trial with treatment as a fixed effect with six levels (bleach [n=215], bleach water [n=212], water bath [n=199], gibberellic acid [n=200], auxin [n=217], ethylene [n=219]), while controlling for seed depth as a continuous fixed effect. For some levels of the treatment fixed effect, no seeds germinated, resulting in a separation problem [1]. Thus, we fit the binomial regression using the package *brglm* (version 0.6.2 [9]) which implements a bias reduction strategy to improve inference from models fit to data with complete separation. We plotted visualized differences in treatment means using the package *emmeans* (version 1.4.7 [11]).

There were not appreciable differences in the effects of individual treatments on germination success (Fig S2): all pre-treatments resulted in low germination success. Given this result, we pooled the pre-treated seeds into one category in the full hierarchical model, such that the fixed effect had two levels: pre-treated or not pre-treated.

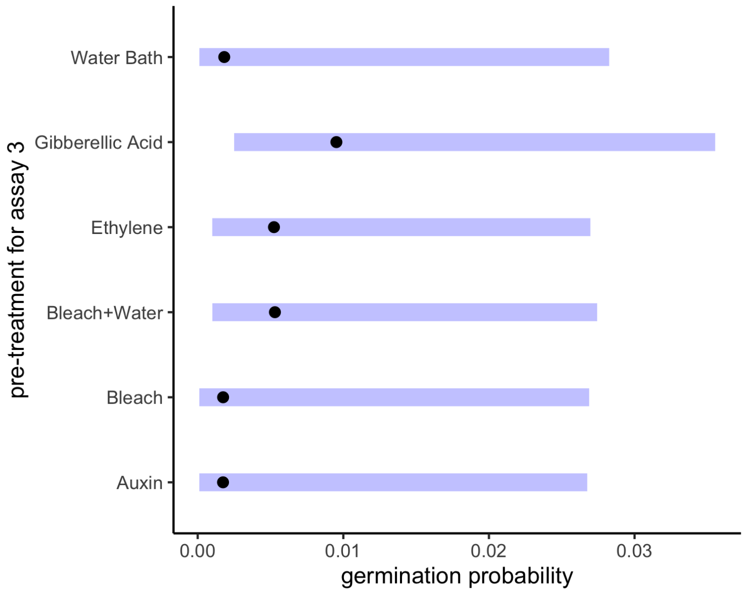


Figure S2: Predicted germination probability in Experiment 3 for seeds that experienced one of six possible pre-treatments. Points represent predicted means and blue bars represent 95% confidence intervals.

## Experiments 4 and 5

Previous work suggests that fluctuating temperature promotes breaking dormancy in seeds of sedge species [14, 8]. Therefore, we planted seeds in petri dishes with moist sterile white construction sand and exposed them to one of three temperature regimes: a constant 30°C, a fluctuation of 27/15°C and a fluctuation of 20/15°C. Temperature was adjusted using digitally controlled seedling heat mats (Hydrofarm, Petaluma, CA, USA) and was aligned with a photoperiod of 15 hours light (high temperature): 9 hours dark (low temperature). A subset of petri dishes was assigned to an all-dark treatment (wrapped in aluminum foil) to test for the efficacy of the photoperiod.

## Experiments 6 and 7

Tissue culture of the endosperm of seeds was conducted according to Lauzer (2004). We extracted embryos from 25 seeds and kept 25 seeds intact for a fluctuating temperature and sand treatment, the most successful of previous experiments. The seeds were all sterilized with 70% ethanol and 6% sodium hypochlorite (Tween 20 was used as a surfactant). We used a growth medium consisting of Murashige and Skoog salts and vitamins combined with 30 g/L sucrose and 7 g/L agar. We adjusted the pH to 5.8 before autoclaving for 15 minutes at 121°C. We placed 10 mL of media in each sterilized 15 mL test tube then added one seed embryo to each tube on top of the solid media. Each tube was covered with parafilm and incubated at 25°C (±2°C) with a photoperiod of 15 light: 9 dark [10]. Petri dishes were kept next to the tissue culture tubes with some subjected to the same 25°C temperature with some subjected to fluctuating temperature of 27/15°C.

## Experiments 8 and 9

All seeds were planted in a 1:1 sterile sand and Jiffy Organic Seed Starting Mix (Jiffy, Oslo, Norway). Seeds were placed in the soil mixture in 3" square plastic planters placed in a deep plastic tray, which was flooded with tap water such that the water level in the tray was at approximately 1 cm below the soil surface. Seeds were then placed in a Conviron CMP 6010 growth chamber with the photoperiod regime set at 12 hours of daylight, 12 hours dark, coinciding with a temperature regime of 27°C during daytime and 15°C at night. Seeds were checked every other day for germination or death (indicated by mold) and water levels were kept at an inch below the top of the pot. Seeds were left in the chamber for 14 to 34 days before being removed. Ungerminated seeds were then collected, placed in labelled plastic tubes in de-ionized water, and stored in a refrigerator.

## Experiments 10-13

These experiments followed the same protocols as Experiments 8 and 11, except seeds were potted in seed germination trays, where each seed was placed in a single 1 inch by 1-inch

cell filled with a 1:1 sand and seed starter mix medium. Germination trials ranged from 22 to 117 days.

| Expt | Year | Seed decade range | Media | Photoperiod | Temperature(°C) | Pre-treatment |
|---|---|---|---|---|---|---|
| 1 | 2003 | 1880-2000 | sand/soil | 15/9* | 30 | none |
| 2 | 2004 | 1830-2000 | sand/soil | 15/9 | 30 | none |
| 3 | 2010 | 1860-2010 | sand/soil | 15/9 | 30 | bleach-water, bleach, water, auxin, etephon, gibberellic acid, none |
| 4 | 2010 | 1930-2000 | sand | 15/9, dark | 27/15, 20/15, 30 | none |
| 5 | 2012 | 1840-1960 | sand | 15/9 | 27/15 | none |
| 6 | 2013 | 1840-2010 | sand, growth media | 15/9 | 27/15, 25 | none |
| 7 | 2013 | 1860-2010 | sand, growth media | 15/9 | 27/15, 25 | none |
| 8 | 2017 | 1770-2020 | sand/soil | 12/12 | 27/15 | none |
| 9 | 2017 | 1780-2020 | sand/soil | 12/12 | 27/15 | none |
| 10 | 2017 | 1780-2020 | sand/soil | 12/12 | 27/15 | none |
| 11 | 2018 | 1800-2000 | sand/soil | 12/12 | 27/15 | none |
| 12 | 2018 | 1820-2010 | sand/soil | 12/12 | 27/15 | none |
| 13 | 2019 | 1900-2020 | sand/soil | 12/12 | 27/15 | none |

Table S1: Methods for the thirteen germination experiments conducted from 2003 to 2019. "Year" is the year during which the experiments were conducted. "Seed decade range" corresponds to the predicted decade that a seed was buried in sediment based on the statistical model. "Media" is the type of growth medium used: soil, sand, or tissue culture media. "Photoperiod" corresponds to the number of light hours and dark hours. "Temperature" corresponds to the °C at which the experimental chamber was kept; two numbers signify the fluctuating temperature that corresponded with the photoperiod. "Pre-treatment" signifies the type of pre-treatment used on seeds before a germination trial. *photoperiod for Experiment 1 was 24 hours light for the first 6 days of the trial.

# 3 Results

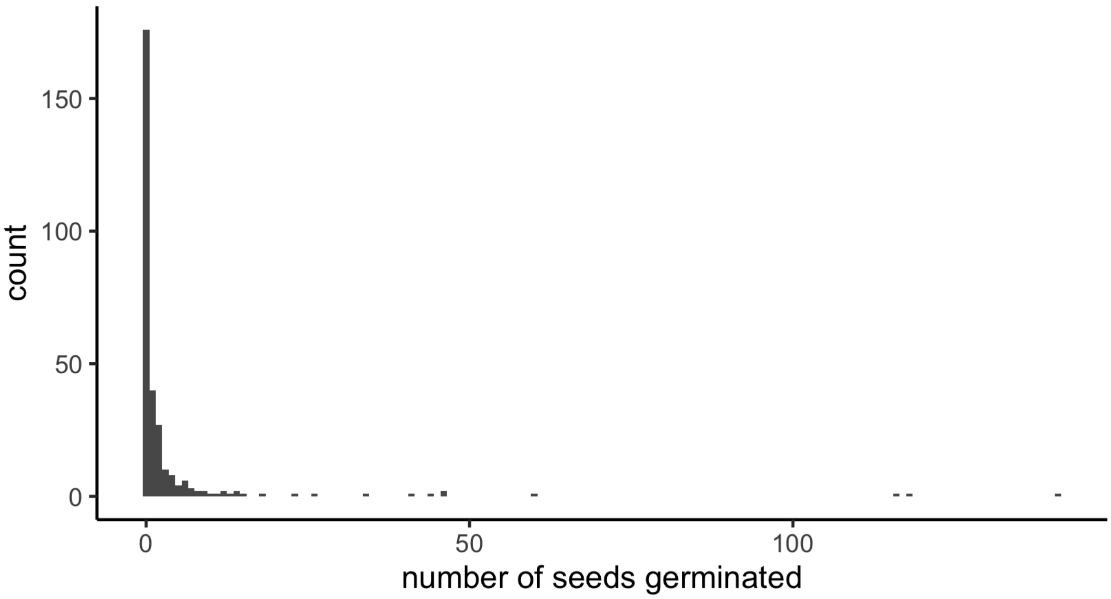


Figure S3: Distribution of raw germination trial data. Each count represents a unique germination trial across seed provenance, temperature, media, photoperiod and pre-treatment (n = 298).

| Model | Likelihood | ZI | OD | WAIC | LOO |
|---|---|---|---|---|---|
| 1 | binomial | - | - | 1084.3 | 1088.1 |
| 2 | zero-inflated binomial | X | - | 1030.4 | 1034.0 |
| 3 | beta-binomial | - | X | 771.9 | 773.2 |
| 4 | zero-inflated beta-binomial | X | X | 773.0 | 774.4 |

Table S2: Comparison of four models fit to germination trial data. ZI = zero-inflation accounted for in the model and OD = overdispersion accounted for in the model. Lower values of WAIC and LOO indicate better model fit.

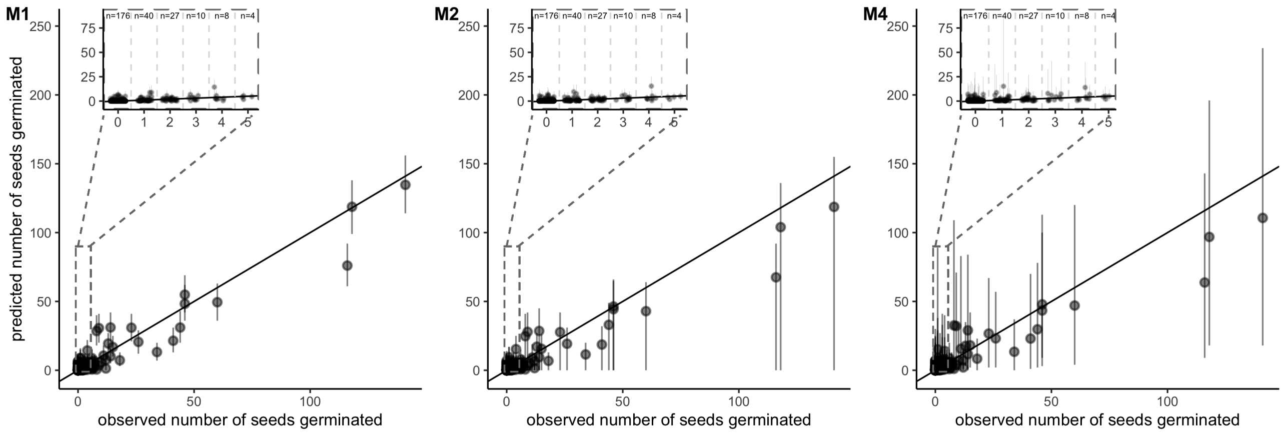


Figure S4: Observed versus predicted plots for Model 1 (binomial likelihood), Model 2 (zero-inflated binomial likelihood), and Model 4 (zero-inflated beta-binomial likelihood). Points represent a unique germination trial across seed provenance, temperature, media, photoperiod, and pre-treatment (n = 298). Bars represent 95% confidence intervals around predicted means from posterior predictive distributions. The inset graph highlights the high density of points where there were five or fewer germinants observed in a trial. Confidence bands for Models 1 and 2 are narrower than in Model 3 (Figure 3a) and Model 4 indicating overconfidence in model parameters.

| Variable | Description | Model 1 | Model 2 | Model 3 | Model |
|---|---|---|---|---|---|
| $y_{a-f}$ | Number of germinated seeds | X | X | X | X |
| $z_{a-f}$ | Number of seeds planted | X | X | X | X |
| $\boldsymbol{x}_{a-f}$ | Regression covariates | X | X | X | X |
| $z_{a-f}$ | Seed age latent variable | X | X | X | X |
| $\beta_0$ | Global intercept for binomial regression | X | X | X | X |
| $\beta_1$ | Seed age regression coefficient for binomial regression | X | X | X | X |
| $\beta_2$ | Media 1 regression coefficient | X | X | X | X |
| $\beta_3$ | Media 2 regression coefficient | X | X | X | X |
| $\beta_4$ | Temperature 1 regression coefficient | X | X | X | X |
| $\beta_5$ | Temperature 2 regression coefficient | X | X | X | X |
| $\beta_6$ | Temperature 3 regression coefficient | X | X | X | X |
| $\beta_7$ | Pre-treatment regression coefficient | X | X | X | X |
| $\beta_8$ | Photoperiod 1 regression coefficient | X | X | X | X |
| $\beta_9$ | Photoperiod 2 regression coefficient | X | X | X | X |
| $\alpha_f$ | Seed provenance random effect | X | X | X | X |
| $\sigma_q$ | Standard deviation for seed provenance random effect | X | X | X | X |
| $\boldsymbol{\gamma}$ | Regression coefficients for seed age calibration model | X | X | X | X |
| $\sigma_p$ | Residual standard deviation for seed age calibration model | X | X | X | X |
| $\pi_{a-f}$ | Latent variable for zero-inflated model | - | X | - | X |
| $\xi_0$ | Intercept for Bernoulli regression | - | X | - | X |
| $\xi_1$ | Seed age regression coefficient for Bernoulli regression | - | X | - | X |
| $\phi$ | Overdispersion parameter for beta-binomial model | - | - | X | X |
| $p_{a-f}$ | Latent variable for seed germination probability in beta-binomial model | - | - | X | X |

Table S3: Data and parameters for hierarchical regression model specifications. Categorical covariates (e.g. Media) were represented in the model using dummy coding such that there are k-1 regression coefficients for k levels of a covariate.

| Subscript | Indexing variable | # unique levels |
|---|---|---|
| a | seed depth | 75 |
| b | media | 3 |
| c | temperature | 4 |
| d | pre-treatment | 2 |
| e | photoperiod | 3 |
| f | seed provenance | 11 |

Table S4: Indices for posterior and joint distributions of the four hierarchical models.

# 4 Model Specification

## Model 1 - Binomial likelihood

### Posterior and joint distribution

$$
\begin{aligned}
[\boldsymbol{\beta}, \alpha_f, \boldsymbol{\gamma}, z_{a-f}, \sigma_p^2, \sigma_q^2] \propto & \prod_{a=1}^{D}\prod_{b=1}^{3}\prod_{c=1}^{4}\prod_{d=1}^{2}\prod_{e=1}^{3}\prod_{f=1}^{11} \text{binomial}(y_{a-f}|n_{a-f}, f(\boldsymbol{\beta}, \alpha_f, z_{a-f}, \boldsymbol{x}_{a-f}))\times \\
& \text{normal}(z_{a-f}|g(\boldsymbol{\gamma}, x_{1,a-f}), \sigma_p^2) \times \text{normal}(\alpha_f|0, \sigma_q^2)\times \\
& \text{multivariate normal}\left(\boldsymbol{\gamma}\middle| \begin{bmatrix} -4.90 \\ 4.12 \\ 0.06 \end{bmatrix}, \begin{bmatrix} 13.63 & -1.86 & 0.05 \\ -1.86 & 0.36 & -0.01 \\ 0.05 & -0.01 & 3.63e^{-4} \end{bmatrix}\right)\times \\
& \text{gamma}(\sigma_p|73.79, 9.08)\times \\
& \text{normal}(\beta_0|0,10) \times \text{normal}(\beta_1|0,10) \times \text{normal}(\beta_2|0,10) \times \text{normal}(\beta_3|0,10)\times \\
& \text{normal}(\beta_4|0,10) \times \text{normal}(\beta_5|0,10) \times \text{normal}(\beta_6|0,10) \times \text{normal}(\beta_7|0,10)\times \\
& \text{normal}(\beta_8|0,10) \times \text{normal}(\beta_9|0,10)\times \\
& \text{inverse gamma}(\sigma_q^2|0.001, 0.001)
\end{aligned}
$$

$$
\begin{aligned}
f(\boldsymbol{\alpha}, \boldsymbol{\beta}, z_{a-f}, \boldsymbol{x}_{a-f}) =& \text{inverse logit}(\beta_0 + \beta_1 z_{a-f} + \beta_2 x_{2,a-f} + \beta_3 x_{3,a-f} + \\
& \beta_4 x_{4,a-f} + \beta_5 x_{5,a-f} + \beta_6 x_{6,a-f} + \beta_7 x_{7,a-f} + \\
& \beta_8 x_{8,a-f} + \beta_9 x_{9,a-f} + \alpha_f) \\
g(\boldsymbol{\gamma}, x_{1,a-f}) =& \gamma_0 + \gamma_1 x_{1,a-f} + \gamma_2 x_{1,a-f}^2
\end{aligned}
$$

## Model 2 - Zero-inflated binomial likelihood

$$
\begin{aligned}
[\boldsymbol{\beta}, \alpha_f, \boldsymbol{\gamma}, \boldsymbol{\xi}, z_{a-f}, \sigma_p^2, \sigma_q^2, \sigma_r^2, \pi_{a-f}] \propto & \prod_{a=1}^{D}\prod_{b=1}^{3}\prod_{c=1}^{4}\prod_{d=1}^{2}\prod_{e=1}^{3}\prod_{f=1}^{11} \text{binomial}(y_{a-f}|n_{a-f}, f(\boldsymbol{\beta}, \alpha_f, z_{a-f}, \boldsymbol{x}_{a-f}))^{\pi_{a-f}}\times \\
& 1_{\{y_{a-f}=0\}}^{1-\pi_{a-f}} \times \text{Bernoulli}(\pi_{a-f}|h(\boldsymbol{\xi}, z_{a-f}))\times \\
& \text{normal}(z_{a-f}|h(\boldsymbol{\gamma}, x_{1,a-f}), \sigma_p^2) \times \text{normal}(\alpha_f|0, \sigma_q^2)\times \\
& \text{multivariate normal}\left(\boldsymbol{\gamma}\middle| \begin{bmatrix} -4.90 \\ 4.12 \\ 0.06 \end{bmatrix}, \begin{bmatrix} 13.63 & -1.86 & 0.05 \\ -1.86 & 0.36 & -0.01 \\ 0.05 & -0.01 & 3.63e^{-4} \end{bmatrix}\right)\times \\
& \text{gamma}(\sigma_p|73.79, 9.08)\times \\
& \text{normal}(\beta_0|0,10) \times \text{normal}(\beta_1|0,10) \times \text{normal}(\beta_2|0,10)\times \\
& \text{normal}(\beta_3|0,10) \times \text{normal}(\beta_4|0,10) \times \text{normal}(\beta_5|0,10)\times \\
& \text{normal}(\beta_6|0,10) \times \text{normal}(\beta_7|0,10) \times \text{normal}(\beta_8|0,10)\times \\
& \text{normal}(\beta_9|0,10) \times \text{normal}(\xi_0|0,10) \times \text{normal}(\xi_1|0,10)\times \\
& \text{inverse gamma}(\sigma_q^2|0.001, 0.001)
\end{aligned}
$$

$$
\begin{aligned}
f(\boldsymbol{\alpha}, \boldsymbol{\beta}, z_{a-f}, \boldsymbol{x}_{a-f}) =& \text{inverse logit}(\beta_0 + \beta_1 z_{a-f} + \beta_2 x_{2,a-f} + \beta_3 x_{3,a-f} + \\
& \beta_4 x_{4,a-f} + \beta_5 x_{5,a-f} + \beta_6 x_{6,a-f} + \beta_7 x_{7,a-f} + \\
& \beta_8 x_{8,a-f} + \beta_9 x_{9,a-f} + \alpha_f) \\
g(\boldsymbol{\gamma}, x_{1,a-f}) =& \gamma_0 + \gamma_1 x_{1,a-f} + \gamma_2 x_{1,a-f}^2 \\
h(\boldsymbol{\xi}, z_{a-f}) =& \text{inverse logit}(\xi_0 + \xi_1 z_{a-f})
\end{aligned}
$$

# Model 3 - Beta-binomial likelihood

$$
\begin{aligned}
[\boldsymbol{\beta}, \alpha_f, \boldsymbol{\gamma}, z_{a-f}, \sigma_p^2, \sigma_q^2, \phi] \propto & \prod_{a=1}^{D}\prod_{b=1}^{3}\prod_{c=1}^{4}\prod_{d=1}^{2}\prod_{e=1}^{3}\prod_{f=1}^{11} \text{binomial}(y_{a-f}|n_{a-f}, p_{a-f})\times \\
& \text{beta}(p_{a-f}|f(\boldsymbol{\alpha}, \boldsymbol{\beta}, z_{a-f}, \boldsymbol{x}_{a-f})\phi, (1 - f(\boldsymbol{\alpha}, \boldsymbol{\beta}, z_{a-f}, \boldsymbol{x}_{a-f})\phi) \\
& \text{normal}(z_{a-f}|g(\boldsymbol{\gamma}, x_{1,a-f}), \sigma_p^2) \times \text{normal}(\alpha_f|0, \sigma_q^2)\times \\
& \text{multivariate normal}\left(\boldsymbol{\gamma}\middle| \begin{bmatrix} -4.90 \\ 4.12 \\ 0.06 \end{bmatrix}, \begin{bmatrix} 13.63 & -1.86 & 0.05 \\ -1.86 & 0.36 & -0.01 \\ 0.05 & -0.01 & 3.63e^{-4} \end{bmatrix}\right)\times \\
& \text{gamma}(\sigma_p|73.79, 9.08)\times \\
& \text{normal}(\beta_0|0, 10) \times \text{normal}(\beta_1|0, 10) \times \text{normal}(\beta_2|0, 10)\times \\
& \text{normal}(\beta_3|0, 10) \times \text{normal}(\beta_4|0, 10) \times \text{normal}(\beta_5|0, 10)\times \\
& \text{normal}(\beta_6|0, 10) \times \text{normal}(\beta_7|0, 10) \times \text{normal}(\beta_8|0, 10)\times \\
& \text{normal}(\beta_9|0, 10) \times \text{gamma}(\phi|0.01, 0.01)\times \\
& \text{inverse gamma}(\sigma_q^2|0.001, 0.001)
\end{aligned}
$$

$$
\begin{aligned}
f(\boldsymbol{\alpha}, \boldsymbol{\beta}, z_{a-f}, \boldsymbol{x}_{a-f}) =& \text{inverse logit}(\beta_0 + \beta_1 z_{a-f} + \beta_2 x_{2,a-f} + \beta_3 x_{3,a-f} + \\
& \beta_4 x_{4,a-f} + \beta_5 x_{5,a-f} + \beta_6 x_{6,a-f} + \beta_7 x_{7,a-f} + \\
& \beta_8 x_{8,a-f} + \beta_9 x_{9,a-f} + \alpha_f) \\
g(\boldsymbol{\gamma}, x_{1,a-f}) =& \gamma_0 + \gamma_1 x_{1,a-f} + \gamma_2 x_{1,a-f}^2
\end{aligned}
$$

## Model 4 - Zero-inflated beta-binomial likelihood

$$
\begin{aligned}
[\boldsymbol{\beta}, \alpha_f, \boldsymbol{\gamma}, \boldsymbol{\xi}, z_{a-f}, \sigma_p^2, \sigma_q^2, \sigma_r^2, \phi, \pi_{a-f}] \propto & \prod_{a=1}^{D}\prod_{b=1}^{3}\prod_{c=1}^{4}\prod_{d=1}^{2}\prod_{e=1}^{3}\prod_{f=1}^{11} \text{binomial}(y_{a-f}|n_{a-f}, p_{a-f})^{\pi_{a-f}} \times \\
& 1_{\{y_{a-f}=0\}}^{1-\pi_{a-f}} \times \text{Bernoulli}(\pi_{a-f}|h(\boldsymbol{\xi}, z_{a-f})) \times \\
& \text{beta}(p_{a-f}|f(\boldsymbol{\beta}, \alpha_f, z_{a-f}, \boldsymbol{x}_{a-f})\phi, (1 - f(\boldsymbol{\beta}, \alpha_f, z_{a-f}, \boldsymbol{x}_{a-f})\phi) \\
& \text{normal}(z_{a-f}|h(\boldsymbol{\gamma}, x_{1,a-f}), \sigma_p^2) \times \\
& \text{normal}(\alpha_f|0, \sigma_q^2) \times \\
& \text{multivariate normal}\left(\boldsymbol{\gamma}\middle| \begin{bmatrix} -4.90 \\ 4.12 \\ 0.06 \end{bmatrix}, \begin{bmatrix} 13.63 & -1.86 & 0.05 \\ -1.86 & 0.36 & -0.01 \\ 0.05 & -0.01 & 3.63e^{-4} \end{bmatrix}\right) \times \\
& \text{gamma}(\sigma_p|73.79, 9.08) \times \\
& \text{normal}(\beta_0|0, 10) \times \text{normal}(\beta_1|0, 10) \times \text{normal}(\beta_2|0, 10) \times \\
& \text{normal}(\beta_3|0, 10) \times \text{normal}(\beta_4|0, 10) \times \text{normal}(\beta_5|0, 10) \times \\
& \text{normal}(\beta_6|0, 10) \times \text{normal}(\beta_7|0, 10) \times \text{normal}(\beta_8|0, 10) \times \\
& \text{normal}(\beta_9|0, 10) \times \\
& \text{normal}(\xi_0|0, 10) \times \text{normal}(\xi_1|0, 10) \times \\
& \text{gamma}(\phi|0.01, 0.01) \times \\
& \text{inverse gamma}(\sigma_q^2|0.001, 0.001)
\end{aligned}
$$

$$
\begin{aligned}
f(\boldsymbol{\alpha}, \boldsymbol{\beta}, z_{a-f}, \boldsymbol{x}_{a-f}) =& \text{inverse logit}(\beta_0 + \beta_1 z_{a-f} + \beta_2 x_{2,a-f} + \beta_3 x_{3,a-f} + \\
& \beta_4 x_{4,a-f} + \beta_5 x_{5,a-f} + \beta_6 x_{6,a-f} + \beta_7 x_{7,a-f} + \\
& \beta_8 x_{8,a-f} + \beta_9 x_{9,a-f} + \alpha_f) \\
g(\boldsymbol{\gamma}, x_{1,a-f}) =& \gamma_0 + \gamma_1 x_{1,a-f} + \gamma_2 x_{1,a-f}^2 \\
h(\boldsymbol{\xi}, z_{a-f}) =& \text{inverse logit}(\xi_0 + \xi_1 z_{a-f})
\end{aligned}
$$

# References


[1] Albert, A., and Anderson, J. A. On the existence of maximum likelihood estimates in logistic regression models. *Biometrika 71*, 1 (1984), 1–10.

[2] Appleby, P. G., and Oldfield, F. The calculation of lead-210 dates assuming a constant rate of supply of unsupported 210pb to the sediment. *Catena 5*, 1 (1978), 1–8.

[3] Bürkner, P.-C., and Buerkner, M. P.-C. Package 'brms'.

[4] Guan, L. M., and Scandalios, J. G. Catalase gene expression in response to auxin-mediated developmental signals. *Physiologia Plantarum 114*, 2 (2002), 288–295.

[5] Hairston Jr, N. G., and Kearns, C. M. Temporal dispersal: ecological and evolutionary aspects of zooplankton egg banks and the role of sediment mixing. *Integrative and Comparative Biology 42*, 3 (2002), 481–491.

[6] Kearney, M. S. Sea-level change during the last thousand years in chesapeake bay. *Journal of Coastal Research* (1996), 977–983.

[7] Kearney, M. S., Stevenson, J. C., and Ward, L. G. Spatial and temporal changes in marsh vertical accretion rates at monie bay: Implications for sea-level rise. *Journal of Coastal Research* (1994), 1010–1020.

[8] Kettenring, K. M., and Galatowitsch, S. M. Temperature requirements for dormancy break and seed germination vary greatly among 14 wetland carex species. *Aquatic Botany 87*, 3 (2007), 209–220.

[9] Kosmidis, I. brglm2: Bias reduction in generalized linear models. *R Package* (2017).

[10] Lauzer, D. In vitro embryo culture of scirpus acutus muhl. *Plant cell, tissue and organ culture 76*, 1 (2004), 91–95.

[11] Lenth, R., Singmann, H., Love, J., Buerkner, P., and Herve, M. Emmeans: Estimated marginal means, aka least-squares means. *R package version 1*, 1 (2018), 3.

[12] Mudd, S. M., Howell, S. M., and Morris, J. T. Impact of dynamic feedbacks between sedimentation, sea-level rise, and biomass production on near-surface marsh stratigraphy and carbon accumulation. *Estuarine, Coastal and Shelf Science 82*, 3 (2009), 377–389.

[13] Norton, C. R. The use of gibberellic acid, ethephon and cold treatment to promote germination of rhus typhina l. seeds. *Scientia horticulturae 27*, 1-2 (1985), 163–169.

[14] Palmisano, A. effect of salinity on the germination and growth of plants important to wildlife in the gulf coast marshes. *Southeast Ass Game Fish Comm Proc* (1972).

[15] Qu, L., Wang, X., Yang, J., Hood, E., and Scalzo, R. Ethephon promotes germination of echinacea angustifolia and e. pallida in darkness. *HortScience 39*, 5 (2004), 1101–1103.

[16] Summers, J. L., Bernik, B., Saunders, C. J., McLachlan, J. S., and Blum, M. J. A century of genetic variation inferred from a persistent soil-stored seed bank. *Evolutionary applications 11*, 9 (2018), 1715–1731.
